# Efficacy of different treatment strategies in patients with mucopolysaccharidosis: a systematic review and network meta-analysis of randomized controlled trials

**DOI:** 10.1186/s13023-025-03735-y

**Published:** 2025-05-02

**Authors:** Lingling Huang, Jianru Wu, Biyu Tang, Jingying Wu, Fenfang Wei, Hong Qiao Li, Limin Li, Xinru Wang, Bei Wang, Wenyu Wu, Xiang Hong

**Affiliations:** 1 Key Laboratory of Environmental Medicine Engineering, Ministry of Education, School of Public Health, Southeast University, Nanjing, 210009 China; 2 Shenzhen Institute of Pharmacovigilance and Risk Management, Shenzhen, 518024 China

**Keywords:** Efficacy, Enzyme replacement therapy, Mucopolysaccharidosis, Network meta-analysis, Randomized controlled trials

## Abstract

This systematic review of randomized controlled trials (RCT) was conducted to evaluate the efficacy of enzyme replacement therapy (ERT) for patients with mucopolysaccharidosis (MPS). We systematically searched PubMed, Embase, Web of Science, and Cochrane databases up to August 22, 2023. Study design, interventions, and outcome data were extracted. Continuous variable random-effects network meta-analysis was performed. The review included 23 studies involving 1,047 people with MPS I–VI. In MPS I, urinary glycosaminoglycan (uGAG) level was significantly reduced in patients who took 2 mg/kg/week pentosan polysulfate (-2.66, 95% confidence interval (CI)[-3.86, -1.46]) compared with those who took 1 mg/kg/week. In MPS II, compared with the placebo group, significant reduction were observed in the uGAG (-270.77, 95% CI[-406.57, -139.71]) and the cerebrospinal fluid (CSF) GAG (-1,385.29, 95% CI[-2493.33, -392.65]). In MPS IV, 6-min walking test (6MWT) (40.82, 95% CI[16.19, 64.92]) and 3-min stair climb test (3MSCT) (16.07, 95% CI[12.16, 21.62]) were significantly increased in patients who took elosulfase alfa at a dose of 4.0 mg/kg/week compared with the placebo group. In MPS VI, recombinant human arylsulfatase B (rhASB) and galsulfase (1.0 mg/kg/week) significantly reduced uGAG aggregation compared with the placebo group (-217, 95% CI[-258, -176]) and galsulfase (2.0 mg/kg/week) group (-286.5, 95% CI[-436.5, -136.5]), respectively. Moreover, most studies had high (34.8%) or unclear (43.5%) risk of bias assessments and confidence assessment were low. ERT alleviated symptoms to some extent, but current evidence was insufficient. Hence, further evidence from large-sample RCT is needed.

**Supplementary Information:**

The online version contains supplementary material available at 10.1186/s13023-025-03735-y.

## Introduction

Mucopolysaccharidosis (MPS) is a hereditary lysosomal storage disorder caused by the defects in lysosomal enzymes responsible for degrading glycosaminoglycans (GAGs). It is a rare disease with an estimated prevalence between 0.27 and 2.67 cases per 1 million [1]. Most forms of MPS manifest in early childhood, follow a chronic and progressive course into adulthood, and are associated with significant morbidity and mortality. Without intervention, severe subtypes often lead to multiple organ involvement and markedly shortened life expectancy [2]. Therefore, it is necessary to attach great importance to these patients and take effective treatments in time to improve their quality of life. Currently, this type of disease can be classified into seven types (I, II, III, IV, VI, VII, and IX) and several subtypes based on the lack of lysosomal enzymes, involving 11 lysosomal enzymes encoded by 11 genes [3]. The incidence, severity of clinical symptoms, and prognosis of different subtypes vary with the type of MPS. However, certain common characteristics in clinical manifestations exist, including short stature, special facial features, hepatosplenomegaly, joint activity limit, and progressive central nervous system disease such as cognitive impairment [3]. Therefore, we considered these typical clinical manifestations as outcome indicators to evaluate drug efficacy.

However, some children have relatively serious symptoms such as corneal clouding, slower developmental progression, intellectual deficiency, and endochondral ossification impairment [2, 4–9]. These children develop initial symptoms within a year and can generally only survive until about 10 years without timely treatment [5, 7, 10–12], whereas patients with milder forms of the syndrome can survive until adulthood [13–15].

Moreover, the birth prevalence of MPS II is the highest, accounting for 55% of all patients with MPS between 1982 and 2009 in Japan, followed by that of MPS I, III, and IV, whereas MPS VI, VII, and IX were relatively rare [3], with an estimated global prevalence of 0.04–0.28 per 100,000 live births in patients with MPS VII [16] and only four patients are described as MPS IX type so far without any clinical trials being conducted [17, 18]. Hence, we did not evaluate the curative effect on these patients.

To date, no curative therapies exist for MPS. There are three general modalities currently available: gene therapy, hematopoietic stem cell transplantation (HSCT) and enzymatic replacement (ERT), while each is associated with unique challenges and morbidities [19]. Although current ERT treatment can effectively reduce GAG levels or improve quality of life to some extent, the clinical efficacy is not significant, particularly for the limited improvement in the neurological system [20, 21], skeletal symptoms [21, 22], and cardiopulmonary function [9] (as ERT is not easily accessible to bone and brain tissues through systemic circulation) [23]. Furthermore, the treatment effects of ERT with different doses and administration frequencies are inconsistent for different clinical study designs, which is highly controversial. Few studies have summarized and analyzed the efficacy of this disease using the evidence-based medicine method due to the limited number of cases and relevant clinical trials and the large number of disease subtypes. Therefore, we conducted a thorough network meta-analysis on this disease, systematically evaluating the differences in disease efficacy among various treatments for different subtypes. The goal was to identify the most effective therapy for each subtype and provide guidance for clinical practice. Ultimately, we aimed to better address the challenges of clinical medication and offer hope for treatment to patients with MPS.

## Materials and methods

This review was conducted and reported following the Preferred Reporting Items for Systematic Reviews and Meta-Analyses (Appendix 1) and registered in the International Prospective Register of Systematic Reviews database in October 2023 (Registration number: CRD 42023470374).

### Literature search

We searched literature via PubMed, Embase, Cochrane Library, and Web of Science. The search strategy (Appendix 2) included medical subject heading terms and text words related to different types of mucopolysaccharidosis and randomized controlled trials (RCTs). Then, we considered the population, therapies, comparison, and outcome framework. We included literature from January 01, 2000, onward, focusing exclusively on human studies. We did not restrict our review based on dosage, treatment duration, method of administration (intravenous or intrathecal), study design, or language.

### Inclusion and exclusion criteria

The inclusion criteria were as follows: (a) studies conducted in patients with MPS I–VII; (b) analysis of drug treatment efficacy with a follow-up of 12 weeks or longer; and (c) RCTs.

The exclusion criteria were as follows: (a) nondrug therapy; (b) sample size ≤ 1; (c) animal or cell experiments; and (d) non-RCTs.

### Study endpoints

#### Primary outcome

Reduction in GAG levels in the urine (uGAG) from baseline, measured as mcg/mg creatinine.

#### Secondary outcomes

1. Reduction in GAG levels in the cerebrospinal fluid (CSF GAG) from baseline, measured as mcg/mg creatinine.
2. Reduction in mean liver volume (% of body weight) from baseline.
3. Endurance performance or mobility: 6-min walking test (6MWT) and 3-min stair climb test (3MSCT).
4. Respiratory function: percentage of predicted normal FVC (%FVC) and maximum voluntary ventilation (MVV).
5. Cognitive status: The Bayley Scales of Infant and Toddler Development-III (BSID-III) cognitive developmental quotient (DQ) score.

### Interventions

Six types of MPS interventions were used in the study:

1) MPS I: iaronidase and pentosan polysulfate.

2) MPS II: idursulfase and pabinafusp alfa.

3) MPS III: recombinant human heparan -N- sulfatase (rhHNS) and genistein.

4) MPS IV: elosulfase alfa.

5) MPS VI: odiparcil, rhASB, and galsulfase.

6) MPS VII: vestronidase alfa.

### Literature selection and data extraction

Two reviewers screened the search results independently, retrieved full-text studies, and checked inclusion criteria. In case of doubt, a third reviewer was consulted.

Two reviewers independently extracted data from selected studies. The basic characteristics, such as first author, disease type, participants (age and sample size), study design (blinding), details of interventions (drug, dose, and duration), and outcome measures were abstracted and recorded into a pre-made form. Any differences were discussed, and a third reviewer was contacted if consensus was not reached. The study authors were contacted in case of missing or unclear information. We provided summaries of intervention effects for each study by calculating standardized mean differences for continuous outcomes.

### Risk-of-bias assessment

Two reviewers assessed the studies included in the meta-analysis for the risk of bias based on the Cochrane Collaboration’s Revised Cochrane Risk-of-Bias tool 2 (Rob2). Five domains were evaluated using this tool: risk of bias in the randomization process, risk of bias due to deviations from established intervention measures, bias in missing outcome data, bias in outcome measurement, and risk of bias in selection of the reported result. The reported treatment effects of the studies were evaluated for each domain as low risk, some concerns, or high risk. The overall bias was classified as “low risk of bias” if all domains were rated as low. It was characterized as “some concerns” if no high risk of bias existed and all domains were rated as low or some concerns. Conversely, it was classified as “high risk of bias” if one or more domains were rated as high risk of bias. Two reviewers independently performed the risk-of-bias assessment. Disagreements were resolved by consensus; failing that, a third reviewer (Wenyu Wu) made the final decision.

### Assessing the certainty of the evidence

The Confidence in Network Meta-Analysis (CINeMA) system, a free and open-source CINeMA software (https://cinema.ispm.unibe.ch/), was used to evaluate the credibility of each outcome in the network meta-analysis, which was based on the Grading of Recommendations, Assessment, Development, and Evaluation and simplified the evaluation process. Six domains were evaluated, including: (a) within-study bias, (b) reporting bias, (c) indirectness, (d) imprecision, (e) heterogeneity, and (f) incoherence. Each domain was rated at three levels: “no concerns,” “some concerns,” or “major concerns.”

### Statistical analysis

We performed Bayesian network meta-analyses to compare the effects of different therapeutic drugs. This approach calculated the posterior distribution of the parameters by updating prior information with the available data and was more common than frequentist approaches [24]. Markov chains were used to generate samples. Model convergence was assessed using the Brooks-Gelman-Rubin plots method [25, 26]. Global heterogeneity was assessed on the bias of the magnitude of heterogeneity variance parameter estimated from the network meta-analyses models [27, 28]. All included interventions were considered for synthesizing the data. However, if the treatments could not form a connected loop with other interventions, they were not compared and analyzed in the network meta-analysis. A node-splitting method was used to examine the inconsistency between direct and indirect comparisons when a loop connecting three arms existed [29, 30]. The ranking probabilities for all treatments were estimated, and a treatment hierarchy using the probability of being the best treatment was obtained. This process was performed using the surface under the cumulative ranking curve (SUCRA) to rank the treatments based on efficacy; the greater the SUCRA score, the more effective the drug [31, 32]. The statistical analysis was performed using Stata 14.0 and RStudio 4.3.1 with the package “GEMTC” V.1.0. The data that could be merged were analyzed using the Bayesian random-effects model. For continuous data, the pooled estimated mean difference (MD) (95% confidence interval) for different outcomes in different types of patients with MPS was as follows: MD = 0 indicated no difference between the two groups; MD < 0 indicated that the former group had a smaller value, and MD > 0 indicated that the group had a larger value.

## Results

### Search results

**Fig. 1:**
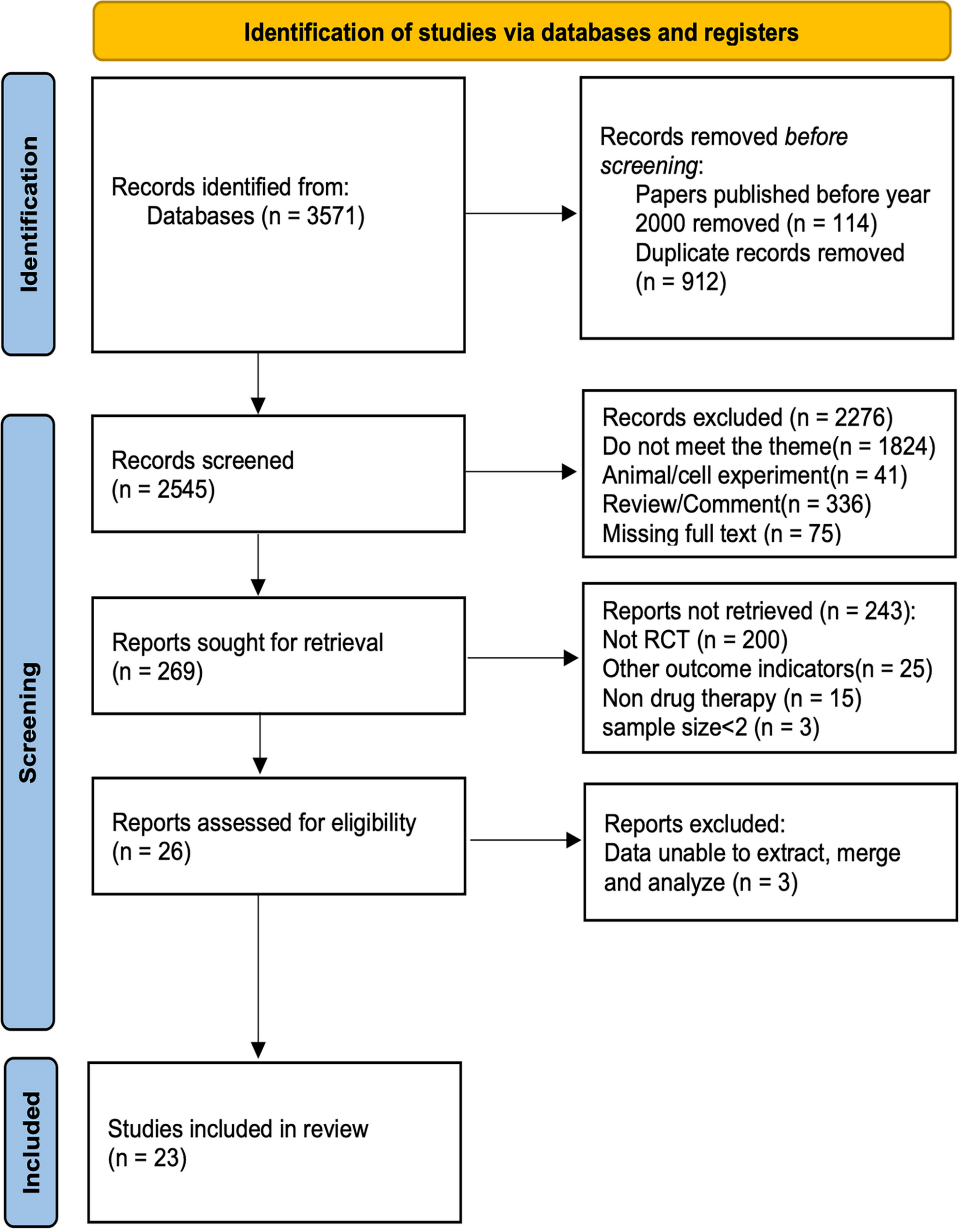
Flow diagram outlining the selection process

Initially, 3,571 studies were identified, of which 912 duplicate studies and 114 studies published before year 2000 were removed. After reviewing titles and abstracts, 2,276 studies were excluded and the remaining were considered potentially eligible for full-text screening. Of the studies reviewed in full, 203 were excluded for not being RCTs. Then, 43 studies were excluded from selection due to the lack of treatment using chemical drugs, nonconforming outcome indicators, and insufficient sample size. Finally, 26 studies that met the requirements were included in this network meta-analysis. Finally, 23 studies were retained in this meta as three clinical trials of them currently reported were long-term extension studies with dynamic enrollment and it was hard to extract, merge. and analyze the data. The flow chart of the study selection process and studies considered for inclusion is shown in Fig. 1.

### Characteristics of the included studies

We provided summaries of the findings from the included studies, structured around the type of intervention, target population characteristics, study features, type of outcome, intervention dose, and frequency. The main characteristics of the included studies are shown in Appendix 3. The average time of treatment duration was 37 ± 18.79 weeks (the 18th study used a natural population cohort without treatment as a control, leading to cause bias, and hence was not included).

### Efficacy outcomes

A total of 23 studies were included. The data in seven studies were not included in the combined statistics by classification and described individually due to the inability to merge data. Nineteen studies reported the changes in GAG levels, and three of them were based on the CSF level. Seventeen studies analyzed the effect on endurance performance, eleven were on 6MWT, and six were on 3MSCT. Seven studies evaluated the respiratory function.

**Fig. 2:**
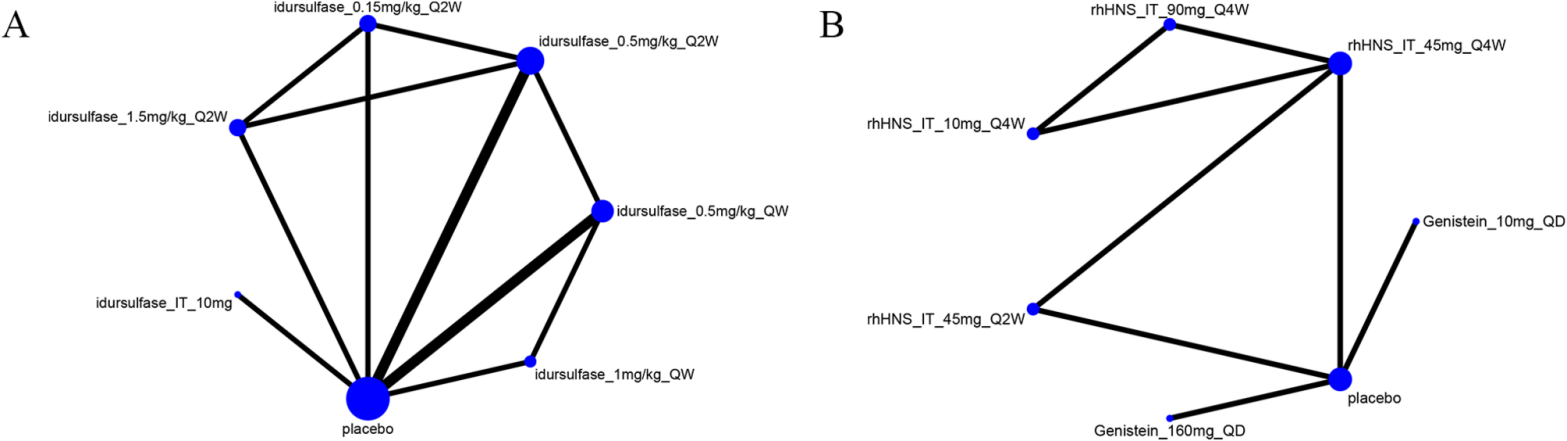
Network plot of eligible comparison for the primary outcome uGAG level: (**A**) MPS II and (**B**) MPS III. The dots represent treatments involved, and size means the sample size. The lines between the dots represent comparisons between treatments, and their thickness means the sample size

Figure 2 shows the network plot for the reduction in uGAG level from baseline with treatments in patients with MPS II and III. The data on MPS I and VI were discarded because they could not be merged. All other network plots for the secondary outcomes are shown in Appendix 4.

#### MPS I

In patients with MPS I, the respiratory function of patients treated with laronidase (0.58 mg/kg/week) increased by 5.6, 95% CI[2.40, 8.80] compared with the placebo group, and their 6MWT was 38.1, 95% CI[10.10, 66.10] longer than the placebo one. Also, 2 mg/kg pentosan polysulfate treatment led to a larger decrease in GAG level in urine compared with 1 mg/kg pentosan polysulfate (-2.66, 95% CI[-3.86, -1.46]) (Table 1).

**Table 1:** Pairwise meta-analysis results of different outcomes in studies unable to merge

0	Outc1ome	No.	Treat 1	Treat 2	Mean Difference(95% Crl)
I	6MWT	1	laronidase_0.58 mg/kg_QW	placebo	**38.1(10.10**,**66.10)**
	FVC	1	laronidase_0.58 mg/kg_QW	placebo	**5.6(2.40**,**8.80)**
	uGAG	2	pentosan_polysulphate_2mg/kg_QW	pentosan polysulphate_1mg/kg_QW	**-2.66(-3.86**,**-1.46)**
II	Liver V	9	pabinafusp_alfa_4.0 mg/kg	pabinafusp_alfa_2.0 mg/kg	**-13.3(-24.3**,**-2.3)**
VI	uGAG	20	galsulfase + odiparcil_1000 mg/day	galsulfase + placebo	**1636.3(1006.3**,**2266.3)**
		20	galsulfase + odiparcil_500 mg/day	galsulfase + placebo	**545.4(365.4**,**725.4)**
		20	odiparcil_1000 mg/day	galsulfase + placebo	**1413.4(453.4**,**2373.4)**
		21	rhASB	placebo	**-217(-258**,**-176)**
		23	galsulfase_1.0 mg/kg_QW	galsulfase_2.0 mg/kg_QW	**-286.5(-436.5**,**-136.5)**
	6MWT	20	galsulfase + odiparcil_1000 mg/day	galsulfase + placebo	-75(-151,1)
		20	galsulfase + odiparcil_500 mg/day	galsulfase + placebo	-27.67(-106.67,51.33)
		20	odiparcil_1000 mg/day	galsulfase + placebo	-30.5(-108.5,47.5)
		22	rhASB_0.2 mg/kg	rhASB_1.0 mg/kg	5(-78,88)
VI	FVC	20	galsulfase + odiparcil_1000 mg/day	galsulfase + placebo	**0.12(0.021**,**0.219)**
		20	galsulfase + odiparcil_500 mg/day	galsulfase + placebo galsulfase + placebo	**0.13(0.031**,**0.229)**
		20	odiparcil_1000 mg/day	placebo	**0.13(0.01**,**0.25)**
		21	rhASB	galsulfase + placebo	-0.01(-0.029,0.129)
	MVV	20	galsulfase + odiparcil_1000 mg/day	galsulfase + placebo	**3.9(2.5**,**5.3)**
		20	galsulfase + odiparcil_500 mg/day	galsulfase + placebo	8.9(-16.1,33.9)
		21	rhASB	placebo	1.9(-1,4.8)

6MWT: 6-min walking test; FVC: forced vital capacity; MVV: maximum voluntary ventilation; rhASB: recombinant human arylsulfatase B; uGAG: urinary glycosaminoglycan; Liver V: liver volumes

#### MPS II

The decrease in uGAG excretion under idursulfase treatment at the doses of 0.5 mg/kg/week, 0.5 mg/kg every other week, 0.15 mg/kg every other week, and 1.5 mg/kg every other week was more than that in the placebo group (-131.23, 95% CI[-219.31, -56.76]; -139.15, 95% CI[-232.4, -55.48]; -149.73, 95% CI[-290.13, -14.41]; -270.77, 95% CI[-406.57, -139.71]). Moreover, 1.5 mg/kg every other week treatment with idursulfase had significantly better effects than 1 mg/kg/week treatment (-166.58, 95% CI[-333.69, -2.46]) and 10 mg idursulfase-IT treatment (-261, 95% CI[-453.26, -75.58]) (Appendix 5 A). Further, 1 and 10 mg idursulfase-IT had significant effects on the CSF GAG excretion compared with placebo (-1385.29, 95% CI[-2493.33, -392.65]; -1030.6, 95% CI[-1847.87, -383.37]) (Appendix 5B). The results were significant on the liver volume reduction (-17.11, 95% CI[-32.12, -2.6]) and 6MWT improvement (54.12, 95% CI[17.93, 89.78]) after treatment with 0.5 mg/kg/week idursulfase compared with the placebo (Appendix 5 C and D). The pulmonary function test FVC outcome indicated that patients treated with 1 mg/kg/week idursulfase achieved better recovery than those who took 0.5 mg/kg every other week (13.63, 95% CI[0.13, 26.78]) and placebo (13.39, 95% CI[2.17, 24.74]) (Appendix 5E). Similarly, the liver volumes decreased more from baseline with pabinafusp alfa at a dose of 4.0 mg/kg compared with 2.0 mg/kg (-13.3,95% CI [-24.3,-2.3]) [33] (Table 1).

#### MPS III

Four types of patients had severe cognitive impairment, and the degradation of heparan sulfate (HS) was retarded despite the lack of different enzymes. The evidence showed that different doses of rhHNS and genistein did not significantly reduce GAG levels in the urine and improve cognitive function, whether globally or in individual studies (Appendix 5 F and G). The results indicated that effective treatment methods were lacking for MPS III patients.

#### MPS IV

RCTs of MPS IV included used keratan sulfate levels in the urine (uKS) from baseline to evaluate GAG aggregation. Therefore, we used this indicator to analyze the uGAG reduction. In this network meta-analysis, no statistically significant difference was found in uKS among different therapies (Appendix 5 H). Both elosulfase alfa doses at 2 or 4 mg/kg/week in patients with MPS IV resulted in a significant increase in 6MWT (26.2, 95% CI[6.92, 46.43]; 40.82, 95% CI[16.19, 64.92]). Patients treated with elosulfase alfa (4.0 mg/kg/week) performed better than those treated with 2 mg/kg every other week in 6MWT (37.73, 95% CI[10.99, 63.81]), whereas the result was not significant for the other dose (2.0 mg/kg/week) (14.67, 95% CI[-0.71, 28.31]) and the efficacy was better for weekly treatment than for biweekly treatments, both with 2.0 mg/kg elosulfase alfa (23.17, 95% CI[1.29, 45.39]) (Appendix 5I). Elosulfase alfa (4 mg/kg/week) was statistically significant in improving 3MSCT compared with the other doses 2.0 mg/kg/week or every other week and the placebo group (14.57, 95% CI[11.16; 18.18]; 16.25, 95% CI[11.82, 21.74]; 16.07, 95% CI[12.16, 21.62], separately). (Appendix 5 J).

#### MPS VI

Individual absolute changes in Table 1 from baseline showed that galsulfase administered separately at 1 mg/kg/week significantly reduced the accumulation of GAG in the urine (-286.5, 95% CI[-436.5, -136.5]) compared with 2 mg/kg/week treatment [34]. Conversely, the uGAG storage remained after taking odiparcil compared with that in the control group (1636.3, 95% CI[1006.3, 2266.3]; 545.4, 95% CI[365.4, 725.4]; 1413.4, 95% CI[453.4, 2373.4]) [35] Nevertheless, odiparcil treatment resulted in an increase in FVC, though only with a small significance (0.12, 95% CI[0.021, 0.219]; 0.13, 95% CI[0.031, 0.229]; 0.13, 95% CI[0.01, 0.25]). In addition, galsulfase combined with 1,000 mg/day odiparcil increased MVV levels compared with that in the control group (3.9, 95% CI[2.5, 5.3]). However, no combination method could increase the 6-min walking distance. This result indicated that the effects of combined administration could not be as good as those of a single administration. Odiparcil might not exert a marked effect in the treatment process and even attenuated the pesticidal effect of galsulfase. Moreover, a previous study [36] showed that another drug rhASB did not improve FVC and MVV, it significantly reduced the excretion of GAG in the urine (-217, 95% CI[-258, -176]) compared with the placebo group.

#### Ranking

Appendix 5 presents the results of the two-by-two comparison between groups drawn to league tables, and Table 2 depicts the SUCRA ranks.

**Table 2:** Ranking results of each treatment in the network

Type	Outcomes	Rank						
		1	2	3	4	5	6	7
II	uGAG	idursulfase_1.5 mg/kg_Q2W	idursulfase_0.15 mg/kg_Q2W	idursulfase_0.5 mg/kg_Q2W	idursulfase_0.5 mg/kg_QW	idursulfase_1mg/kg_QW	idursulfase_IT_10mg	placebo
	CSF GAG	Idursulfase_IT_1mg	Idursulfase_IT_10mg	Idursulfase_IT_30mg	placebo			
	6MWT	idursulfase_0.5 mg/kg_QW	idursulfase_1mg/kg_QW	idursulfase_0.5 mg/kg_Q2W	placebo			
	FVC	idursulfase_1mg/kg_QW	idursulfase_0.5 mg/kg_QW	placebo	idursulfase_0.5 mg/kg_Q2W			
	Liver V	idursulfase_0.5 mg/kg_QW	idursulfase_0.5 mg/kg_Q2W	idursulfase_1.5 mg/kg_Q2W	idursulfase_0.15 mg/kg_Q2W	placebo		
III	uGAG	rhHNS_IT_10mg_Q4W	rhHNS_IT_90mg_Q4W	rhHNS_IT_45mg_Q4W	rhHNS_IT_45mg_Q2W	Genistein_160mg_QD	Genistein_10mg_QD	placebo
	DQ	Genistein_160_mg_QD	rhHNS_IT_45_mg_Q4W	placebo	rhHNS_IT_45_mg_Q2W			
IV	6MWT	elosulfase_alfa_4.0 mg/kg_QW	elosulfase_alfa_2.0 mg/kg_QW	elosulfase_alfa_2.0 mg/kg_Q2W	placebo			
	3MSCT	idursulfase_1.5 mg/kg_Q2W	idursulfase_0.15 mg/kg_Q2W	idursulfase_0.5 mg/kg_Q2W	idursulfase_0.5 mg/kg_QW	idursulfase_1mg/kg_QW	idursulfase_IT_10mg	placebo
	uKS	Idursulfase_IT_1mg	Idursulfase_IT_10mg	Idursulfase_IT_30mg	placebo			

3MSCT: 3-min stair climb test; 6MWT: 6-min walking test; CSF GAG: cerebrospinal fluid glycosaminoglycan; DQ: developmental quotient; FVC: forced vital capacity; uKS: urine keratan sulfate; uGAG: urinary glycosaminoglycan; Liver V: liver volumes

### Model fit, heterogeneity, and inconsistency assessment

The convergence of the models was assessed using the trace and Brooks-Gelman-Rubin diagnosis plots. In each outcome, the density map was normally distributed and the potential scale reduction factor (PSFR) approached 1, indicating that the model had a satisfactory convergence and could predict the data effectively (Appendix 6).

Bayesian framework in the random-effects models was used to verify the consistency. The global inconsistency *I*^2^ values for all outcomes were not more than 20 (Table 3), and the Bayesian *P* values generated by the node-splitting method were above 0.05 (Table 4), which supported the assumption of satisfying consistency between direct and indirect comparisons for all outcomes.

**Table 3:** Analysis of global inconsistency and heterogeneity

Type	Global inconsistency		Global heterogeneity	
	Clinical outcome			
		*I*^*2*^(%)	*I*^*2*^.pair(%)	*I*^*2*^.cons(%)
	uGAG	0	79.60122	57.99318
	CSF GAG	0	2.668657	0
II	6MWT	6	36.38444	0
	FVC	0	0	0
	Liver V	11	98.43523	98.27598
III	uGAG	0	100	0
	DQ	0	0	0
	6MWT	0	2.986462	0
IV	3MSCT	0	28.66967	0
	uKS	20	99.99383	99.99137

3MSCT: 3-min stair climb test; 6MWT: 6-min walking test; CSF GAG: cerebrospinal fluid glycosaminoglycan; DQ: developmental quotient; FVC: forced vital capacity; uKS: urine keratan sulfate; uGAG: urinary glycosaminoglycan; Liver V: liver volumes

**Table 4:** Analysis of comparison inconsistency and heterogeneity

				Inconsistency	Heterogeneity		
Type	Clinical outcome	Treat1	Treat2				
				*p*.value	I2.pair(%)	I2.cons(%)	*p*.value
		idursulfase_0.5 mg/kg_QW	idursulfase_0.5 mg/kg_Q2W	0.62	NA	0.00	0.82
		placebo	idursulfase_0.5 mg/kg_Q2W	0.63	NA	0.00	0.80
	6MWT	idursulfase_1mg/kg_QW	idursulfase_0.5 mg/kg_QW	0.66	NA	0.00	0.82
		placebo	idursulfase_0.5 mg/kg_QW	0.96	36.37	0.00	0.98
		placebo	idursulfase_1mg/kg_QW	0.65	NA	0.00	0.82
		idursulfase_0.5 mg/kg_Q2W	idursulfase_0.15 mg/kg_Q2W	0.48	37.26	96.30	0.68
II		idursulfase_1.5 mg/kg_Q2W	idursulfase_0.15 mg/kg_Q2W	0.98	87.17	76.86	0.99
		placebo	idursulfase_0.15 mg/kg_Q2W	0.37	NA	98.79	0.58
	Liver V	idursulfase_0.5 mg/kg_QW	idursulfase_0.5 mg/kg_Q2W	0.20	NA	90.01	0.47
		idursulfase_1.5 mg/kg_Q2W	idursulfase_0.5 mg/kg_Q2W	0.50	0	96.24	0.69
		placebo	idursulfase_0.5 mg/kg_Q2W	0.57	99.59	98.81	0.72
		placebo	idursulfase_0.5 mg/kg_QW	0.20	NA	90.61	0.47
		placebo	idursulfase_1.5 mg/kg_Q2W	0.43	NA	98.70	0.62
		Idursulfase_IT_1mg	Idursulfase_IT_10mg	0.99	NA	0.00	0.99
		Idursulfase_IT_30mg	Idursulfase_IT_10mg	0.54	38.58	0.00	0.82
	CSF GAG	placebo	Idursulfase_IT_10mg	0.50	0	0.00	0.87
		Idursulfase_IT_30mg	Idursulfase_IT_1mg	0.79	NA	0.00	0.86
		placebo	Idursulfase_IT_1mg	0.75	NA	0.00	0.79
		placebo	Idursulfase_IT_30mg	0.60	NA	0.00	0.66
		idursulfase_0.5 mg/kg_QW	idursulfase_0.5 mg/kg_Q2W	0.85	NA	0.00	0.96
		placebo	idursulfase_0.5 mg/kg_Q2W	0.86	NA	0.00	0.95
	FVC	idursulfase_1mg/kg_QW	idursulfase_0.5 mg/kg_QW	0.78	NA	0.00	0.82
		placebo	idursulfase_0.5 mg/kg_QW	1.00	0	0.00	0.97
		placebo	idursulfase_1mg/kg_QW	0.78	NA	0.00	0.88
		idursulfase_0.5 mg/kg_Q2W	idursulfase_0.15 mg/kg_Q2W	0.92	NA	0.00	0.96
	uGAG	idursulfase_1.5 mg/kg_Q2W	idursulfase_0.15 mg/kg_Q2W	0.99	NA	0.00	1.00
		placebo	idursulfase_0.15 mg/kg_Q2W	0.93	NA	0.00	0.95
		idursulfase_0.5 mg/kg_QW	idursulfase_0.5 mg/kg_Q2W	0.32	NA	45.55	0.77
		idursulfase_1.5 mg/kg_Q2W	idursulfase_0.5 mg/kg_Q2W	0.91	NA	0.00	0.96
		placebo	idursulfase_0.5 mg/kg_Q2W	0.43	0	0.00	0.78
II	uGAG	idursulfase_1mg/kg_QW	idursulfase_0.5 mg/kg_QW	0.43	NA	55.29	0.84
		placebo	idursulfase_0.5 mg/kg_QW	0.93	89.75	79.13	0.99
		placebo	idursulfase_1.5 mg/kg_Q2W	0.91	NA	0.00	0.95
		placebo	idursulfase_1mg/kg_QW	0.43	NA	49.15	0.83
		rhHNS_IT_45mg_Q2W	placebo	1.00	NA	0.00	1.00
		rhHNS_IT_45mg_Q4W	placebo	1.00	NA	0.00	0.99
III	uGAG	rhHNS_IT_45mg_Q4W	rhHNS_IT_10mg_Q4W	1.00	NA	0.00	1.00
		rhHNS_IT_90mg_Q4W	rhHNS_IT_10mg_Q4W	1.00	NA	0.00	1.00
		rhHNS_IT_45mg_Q4W	rhHNS_IT_45mg_Q2W	0.99	NA	0.00	0.99
		rhHNS_IT_90mg_Q4W	rhHNS_IT_45mg_Q4W	1.00	NA	0.00	1.00
III	DQ	rhHNS_IT_45_mg_Q2W	placebo	0.88	0	0.00	0.92
		rhHNS_IT_45_mg_Q4W	placebo	0.91	0	0.00	0.92
		rhHNS_IT_45_mg_Q4W	rhHNS_IT_45_mg_Q2W	0.90	8.46	0.00	0.89
		elosulfase_alfa_2.0 mg/kg_QW	elosulfase_alfa_2.0 mg/kg_Q2W	0.78	NA	0.00	0.97
	3MSCT	placebo	elosulfase_alfa_2.0 mg/kg_Q2W	0.78	NA	0.00	0.97
		placebo	elosulfase_alfa_2.0 mg/kg_QW	0.77	76.11	27.73	0.86
		elosulfase_alfa_2.0 mg/kg_QW	elosulfase_alfa_2.0 mg/kg_Q2W	0.49	NA	99.98	0.58
IV	uKS	placebo	elosulfase_alfa_2.0 mg/kg_Q2W	0.50	NA	99.98	0.58
		placebo	elosulfase_alfa_2.0 mg/kg_QW	0.50	100	99.99	0.60
		elosulfase_alfa_2.0 mg/kg_QW	elosulfase_alfa_2.0 mg/kg_Q2W	0.84	NA	0.00	0.90
	6MWT	placebo	elosulfase_alfa_2.0 mg/kg_Q2W	0.84	NA	0.00	0.89
		placebo	elosulfase_alfa_2.0 mg/kg_QW	0.84	0	0.00	0.87

3MSCT: 3-min stair climb test; 6MWT: 6-min walking test; CSF GAG: cerebrospinal fluid glycosaminoglycan; DQ: developmental quotient; FVC: forced vital capacity; uKS: urine keratan sulfate; uGAG: urinary glycosaminoglycan; Liver V: liver volumes

The global heterogeneity and pairwise comparisons of heterogeneity are shown in Tables 3 and 4. The results indicated heterogeneity in the global comparison of uGAG level and liver volume reduction in MPS II and uKS in MPS IV (*I*^2^ = 57.99%, 98.28%, and 99.99%, respectively), whereas no significant global heterogeneity (*I*^2^ < 50%) was observed among studies of other outcomes in the network meta-analysis. However, the *P* value of heterogeneity in each comparison group was more than 0.05, indicating that the comparisons between two interventions were homogeneous. We found that the inconsistency in the duration of therapy in different studies was a source of heterogeneity in this meta-analysis. We did not implement subgroup analysis or regression tests for different therapies owing to the acceptability of the heterogeneity test.

The publication bias assessment funnel diagrams in different outcomes of the included studies are shown in Appendix 7. The funnel plots were not symmetric. The Egger’s test suggested publication bias in 6MWT in patients with MPS IV (*P* = 0.0210) and in 3MSCT in patients with MPS IV (*P* = 0.0042). It did not indicate publication bias in other outcomes (Table 5).

**Table 5:** The result of Egger’s test for each outcome

Type	Outcome	*p*.value
II	6MWT	0.9018
	Liver V	0.5388
	CSF GAG	0.1631
	FVC	0.619
	uGAG	0.7812
III	DQ	0.126
	uGAG	0.3035
IV	6MWT	**0.0210**
	3MSCT	**0.0042**
	uKS	0.8777

3MSCT: 3-min stair climb test; 6MWT: 6-min walking test; CSF GAG: cerebrospinal fluid glycosaminoglycan; DQ: developmental quotient; FVC: forced vital capacity; uKS: urine keratan sulfate; uGAG: urinary glycosaminoglycan; Liver V: liver volumes

### Risk of bias

**Fig. 3:**
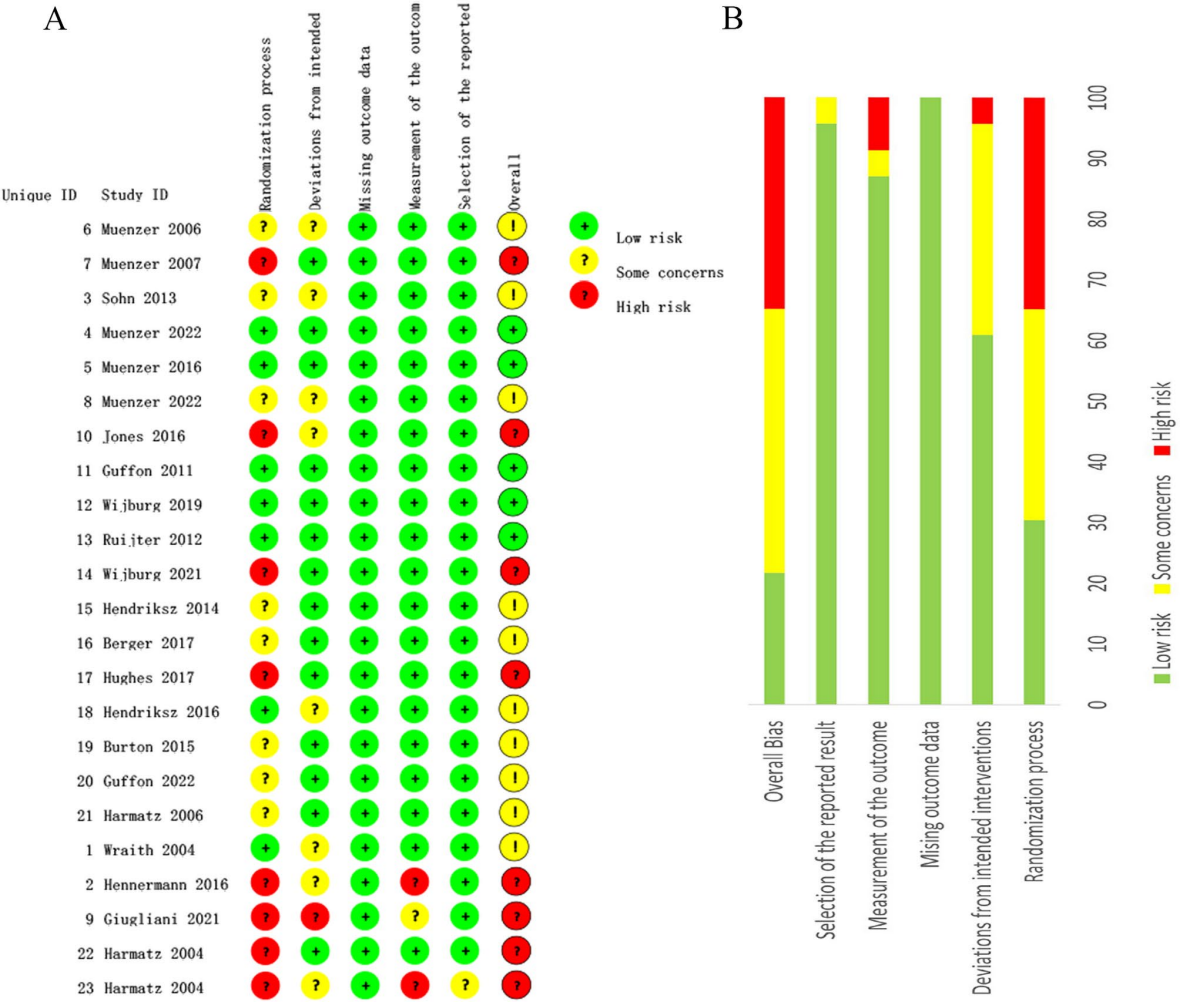
Risk of bias of the RCTs. (**A**) Assessment of risk of bias within each trial. (**B**) Risk-of-bias summary table

The results of the risk of bias of the included studies are reported in Fig. 3. The risk levels of each independent item in the assessment of all included studies were categorized as low, moderate, and high. Their proportions were as follows: overall (21.7%, 43.5%, and 34.8%, respectively); randomization process (30.4%, 34.8%, and 34.8%, respectively); deviations from intended interventions (60.9%, 34.8%, and 4.3%, respectively); missing outcome data (100%, 0%, and 0%, respectively); measurement of the outcome (87.0%, 4.3%, and 8.7%, respectively); and selective reporting bias (95.7%, 4.3%, and 0%, respectively).

### Certainty of evidence

Besides evaluating individual studies with Rob2, we used CINeMA to assess the certainty of evidence. The results varied from high to very low. In patients with MPS II, three comparisons about uGAG levels (Appendix 8 A) and one about CSF GAG level (Appendix 8D) scored high or moderate. In patients with MPS IV, three comparisons about 3MSCT (Appendix 8I) and two about 6MWT (Appendix 8 J) scored high or moderate. Other comparisons scored low. Comprehensive detail on CINeMA is provided in Appendix 8.

## Discussion

This network meta-analysis evaluated the efficacy of ERT treatment for different MPS subtypes, the studies included in this network meta-analysis were comprehensive and of good quality. We discussed the relevant available data on efficacy, and the findings were consistent with the results of other studies [9, 37, 38]. The results indicated that pentosan polysaccharide (2 mg/kg/week), idursulfase (1.5 mg/kg every week), and rhASB or galsulfase (1.0 mg/kg/week) were more effective in reducing uGAG level in patients with MPS I, II, and VI. The treatment with laronidase at a dose of 0.58 mg/kg/week and eosulfase alfa at a dose of 4.0 mg/kg/week showed a better endurance performance for patients with MPS I and IV, respectively. Furthermore, 0.58 mg/kg/week laronidase treatment and 1 mg/kg/week idursulfase treatment led to more significant improvements in relieving respiratory function for patients with MPS I and II, respectively. In patients with MPS II, 1 mg idursulfase-IT had a better effect in reducing CSF GAG levels. After treatment with idursulfase at a dose of 0.5 mg/kg/week, the liver volume decreased more significantly, with a longer 6-min walking distance.

Currently, ERT has become the standard treatment for MPS, but this therapy has several controversial issues. First, patients require long-term supplementation with high dose and frequency, contributing to a high cost [39–47]. Second, the efficacy was not good, and it was difficult to transport enzymes across the blood-brain barrier, limiting the improvement in neurological regression [37]. However, patients with MPS IV without intellectual disabilities usually have more beneficial outcomes with ERT, because ERT does not need to cross the blood-brain barrier to improve their neurological function [48]. Third, it was difficult to reverse most pathological changes if patients already had pathological progression before treatment [48]. Last but not least, even with long-term ERT, some symptoms could be improved, but patients may still have some residual symptoms that require multiple surgeries for adjuvant treatment [45]. Now, new treatment options have emerged, such as substrate deprivation, chaperone therapy, gene therapy, and hematopoietic stem cell therapy [49–57]. However, these options have not yet gained widespread attention because of the lack of systematic quantitative evidence to evaluate their benefits. Therefore, we plan to carry out more quantitative evaluations on the efficacy of novel treatments in the future.

By evaluating different outcomes, we found similarities in results for different MPS therapies. The reduction in GAG and visceral organ size, along with the increase in 6MWT and 3MSCT, were considered effective indicators for ERTs. However, clinically, more commonly used indicators such as left ventricular mass index, ejection fraction, range of motion, and endurance test outcomes were excluded from this meta-analysis. This exclusion was due to the inability to combine these findings with already published results, as only few studies are available on these indicators. Additionally, statistical analyses on neuropsychological tests and quality-of-life assessments were not feasible [43, 58–62]. The trials included in this review used mixed pediatric and adult samples, and they did not report adult data separately. Furthermore, in the early phase of the disease, hematopoietic stem cell transplantation (HSCT) was accepted as another form to treat MPS I, but a single RCT on HSCT was not conducted and the outcomes reported did not meet the inclusion criteria. Therefore, these studies were not included [11, 63, 64].

Considering significant differences in age, severity of disease condition, and duration of treatment, we used the Bayesian random-effects model to assess inconsistency and heterogeneity. The network meta-analysis demonstrated global and pairwise consistency. However, individual studies exhibited significant overall heterogeneity, and significant differences were found in indirect comparisons between different therapies. We should consider that the diversity between study plans and many networks was limited, resulting in low power to detect accurate statistical inconsistency and heterogeneity. Small sample sizes and heterogeneity existed in this network meta-analysis.

Apart from the aforementioned limitations, we did not conduct sensitivity and subgroup analyses, since we did not include enough studies. We intend to include a sufficient number of eligible studies (10 or more) in our future study, and plan to undertake both types of analyses to assess the robustness of the results. Additionally, according to CINeMA, we rated many comparisons as low or extremely low quality. Many trials did not report adequate information about randomization and allocation concealment, and restricted the interpretation of these results.

Keratin sulfate is a polysaccharide only stored in patients with MPS IV. Therefore, we evaluated the uKS levels only in these patients. Progressive mental retardation is a characteristic clinical manifestation especially in patients with MPS III. Therefore, our study focused only on the cognitive level changes in MPS III [18, 58]. The results of this network meta-analysis indicated that, to some extent, ERT treatment could reduce the level of GAG and delay the progression of the disease. However, there remain several unsatisfied needs, specifically, MPS III entirely lacked effective therapies. Further studies are needed to address these challenges and provide better solutions for these patients.

Due to phenotypic and genotypic heterogeneity, some complex diseases were classified into multiple molecular subtypes, posing challenges in implementing systematic reviews. Typically, systematic reviews require strict criteria to evaluate the validity of included studies. However, in rare disease research, limitations such as small sample sizes and scarce randomized controlled trials (RCTs) often prevent the inclusion of sufficient homogeneous, high-quality studies. This constraint inevitably introduces a risk of bias in the results.

Furthermore, RCTs for ultra-rare diseases face significant barriers, including small patient populations, high disease heterogeneity, lack of control groups, slow and phenotypically diverse disease progression, and the absence of validated diagnostic and therapeutic measures. These challenges render RCTs impractical, time-prohibitive, or ethically contentious, creating unavoidable limitations for evidence-based clinical practice. Consequently, the confidence in evidence quality is often low or very low, making definitive conclusions difficult to attain.

In the absence of RCTs, alternative evidence sources—such as Real World Evidence (RWE), long-term follow-up studies, and natural history-controlled studies—can provide critical insights into treatment efficacy and safety. For example, using natural history data as a comparator (e.g., disease progression benchmarks) addresses ethical concerns when placebo controls are inappropriate, such as in critically ill populations. Notably, D Hughes [65] and Christian J Hendriksz [66, 67] utilized the Morquio A Clinical Assessment Program (MorCAP) (MOR-001, NCT00787995), a natural history study, as a control group to evaluate enzyme replacement therapy (ERT) outcomes in MPS IVA. Their results demonstrated that patients treated with the optimal dosing regimen showed significant improvements in 6-minute walk test (6MWT), 3-minute stair climb test (3MSCT), and urinary keratan sulfate (uKS) levels compared to untreated patients from the MorCAP study, both in the intention-to-treat (ITT) and modified per-protocol (MPP) populations.

Meanwhile, long-term outcomes in ultra-rare diseases require sustained monitoring, for which RWE and extended follow-up studies are indispensable. The Morquio A Registry Study (MARS), initiated in 2014, collected 10-year longitudinal data from MPS IVA patients, aiming to assess the long-term effectiveness and safety of elosulfase alfa in real-world clinical settings. To date, this remains the largest and longest follow-up study of MPS IVA patients, providing robust evidence on the sustained benefits of ERT in respiratory function and endurance. Key findings in the study from baseline (pre-ERT) to the final follow-up include: a mean percent change of -52.5% (95% CI: -57.5%, -47.4%; *p* < 0.0001) in uKS; a mean change of -6.1 m (95% CI: -27.6, 15.5) in 6MWT and mean changes of 0.2 L (95% CI: 0.1, 0.2) and 0.3 L (95% CI: 0.2, 0.3) in FEV 1 and FVC. Importantly, no new safety concerns or unexpected drug-related adverse events were observed compared to published clinical trial data [68].

Despite inherent limitations in rare disease research, our network meta-analysis accounted for variables such as dosage and administration frequency to enhance methodological rigor. Compared with other systematic reviews, this provided a clearer understanding of specific treatment administrations. Moreover, this review provided a comprehensive overview of the effects of ERT therapies for different MPS subtypes, and the conclusions were consistent with the findings of RCTs and other meta-analyses, enhancing overall credibility.

## Conclusions

The review suggested a clear and consistent effect of pentosan polysulfate (2 mg/kg/week), idursulfase 1.5 mg/kg every other week, and rhASB or galsulfase (1.0 mg/kg/week) in patients with MPS I, II, and VI, leading to a reduction in uGAG excretion. Similarly, significant improvements in physical and respiratory functions were observed after laronidase (0.58 mg/kg/week), idursulfase (1 mg/kg/week) and elosulfase alfa (4.0 mg/kg/week) treatment. Furthermore, different dosages of idursulfase showed varying degrees of effectiveness in improving different outcomes in patients with MPS II. For example, the reduction in CSF GAG level was better under 1 mg/kg idursulfase-IT treatment. The liver volumes decreased more significantly, and 6-min walking distance was longer when the patients were treated with idursulfase at a dose of 0.5 mg/kg/week. However, treatment that could significantly improve symptoms in patients with MPS III are currently lacking. The main limitations of this network meta-analysis included a lack of included trials, small sample sizes, and methodological deficiencies, resulting in low or extremely low confidence in the evidence and an inability to establish definite conclusions. Therefore, we need to collect more data to obtain clear results for evaluating the effects of different therapies.

## Electronic supplementary material

Below is the link to the electronic supplementary material.

Supplementary Material 1: Appendix 1. PRISMA_2020_checklist

Supplementary Material 2: Appendix 2. Search strings and dates of searches.

Supplementary Material 3: Appendix 3. Included studies.

Supplementary Material 4: Appendix 4. Network plots for each outcome.

Supplementary Material 5: Appendix 5. League tables. The columns present the row drug class compared to the column drug class. The rows present the row drug class compared to the column drug class. The effect estimates are expressed as mean ± standard deviation and 95% confidence intervals. Significant results are in bold. A. UGAG difference in patients with MPS II. B. CSF GAG difference in patients with MPS II. C. Liver volumes difference in patients with MPS II. D. 6MWT difference in patients with MPS II. E. FVC difference in patients with MPS II. F. UGAG difference in patients with MPS III. G. Cognitive DQ score difference in patients with MPS III. H. UKS difference in patients with MPS IV. I. 6MWT difference in patients with MPS IV. J. 3MSCT difference in patients with MPS IV.

Supplementary Material 6: Appendix 6. Model fit.

Supplementary Material 7: Appendix 7. The funnel plot of enrolled trials. A, liver volumes reduction in patients with MPS II. B, CSF GAG in patients with MPS II. C, FVC in patients with MPS II. D, uGAG in patients with MPS II. E, 6MWT in patients with MPS II. F, cognitive DQ score in patients with MPS III. G, uGAG in patients with MPS III. H, 6MWT in patients with MPS IV. I, 3MSC in patients with MPS IV. J, uKS in patients with MPS IV.

Supplementary Material 8: Appendix 8. Evaluation of the Certainty of evidence Using CINEMA Framework, mixed evidence. A. UGAG quality in MPS II. B. 6MWT quality in MPS II. C. FVC quality in MPS II. D. CSF GAG quality in MPS II. E. Liver volumes quality in MPS II. F. UGAG quality in MPS III. G. Cognitive DQ score quality in MPS III. H. UKS quality in MPS IV. I. 3MSCT quality in MPS IV. J. 6MWT quality in MPS IV.

## Data Availability

All data are available in the main text or the Supporting Materials.

## Abbreviations

3MSCT: 3-min stair climb test
6MWT: 6-min walking test
BSID-III: The Bayley Scales of Infant and Toddler Development-III
CI: Confidence interval
CSF: Cerebrospinal fluid
CINeMA: The Confidence in Network Meta-Analysis
DQ: Developmental quotient
ERT: Enzyme replacement therapy
FVC: Forced vital capacity
HS: Heparan sulfate
HSCT: Hematopoietic stem cell transplantation
MVV: Maximum voluntary ventilation
MPS: Mucopolysaccharidosis
PSFR: Potential scale reduction factor
rhHNS: Recombinant human heparan -N- sulfatase
rhASB: Recombinant human arylsulfatase B
RCT: Randomized controlled trial
SUCRA: The surface under the cumulative ranking curve
uKS: Urine keratan sulfate
uGAG: Urinary glycosaminoglycan
