# Supplementary material for: Efficacy of different treatment strategies in patients with mucopolysaccharidosis: a systematic review and network meta-analysis of randomized controlled trials
Source: Orphanet J Rare Dis. 2025 May 2;20:211. doi: 10.1186/s13023-025-03735-y (PMC12049060; doi:10.1186/s13023-025-03735-y)
Supplement: Supplementary file 2 — Supplementary Material 2: Appendix 2. Search strings and dates of searches. [file 13023_2025_3735_MOESM2_ESM.pdf]

Appendix 2 Search strings and dates of searches

Database: PubMed <inception to Aug 22, 2023>

#1	Search:((((((((((((((((((((((((((((((((Mucopolysaccharidosis Is[Title/Abstract]) OR (Mucopolysaccharidosis Type I[Title/Abstract])) OR (Lipochondrodystrophy[Title/Abstract])) OR (Lipochondrodystrophies[Title/Abstract])) OR (Hurler Syndrome[Title/Abstract])) OR (Hurler's Disease[Title/Abstract])) OR (Disease, Hurler's[Title/Abstract])) OR (Hurler's Syndrome[Title/Abstract])) OR (Syndrome, Hurler's[Title/Abstract])) OR (Gargoylism[Title/Abstract])) OR (Gargoylisms[Title/Abstract])) OR (Gargoylism, Hurler Syndrome[Title/Abstract])) OR (Hurler Syndrome Gargoylism[Title/Abstract])) OR (Mucopolysaccharidosis Type Ih[Title/Abstract])) OR (Mucopolysaccharidosis Type Ihs[Title/Abstract])) OR (Type Ih, Mucopolysaccharidosis[Title/Abstract])) OR (Type Ihs, Mucopolysaccharidosis[Title/Abstract])) OR (Hurler Disease[Title/Abstract])) OR (Pfaundler-Hurler Syndrome[Title/Abstract])) OR (Scheie Syndrome[Title/Abstract])) OR (Scheie's Syndrome[Title/Abstract])) OR (Syndrome, Scheie's[Title/Abstract])) OR (Mucopolysaccharidosis Type Is[Title/Abstract])) OR (Mucopolysaccharidosis I-S[Title/Abstract])) OR (Mucopolysaccharidosis I S[Title/Abstract])) OR (Mucopolysaccharidosis V[Title/Abstract])) OR (Mucopolysaccharidosis 5[Title/Abstract])) OR (alpha-L-Iduronidase Deficiency[Title/Abstract])) OR (alpha L Iduronidase Deficiency[Title/Abstract])) OR (alpha-L-Iduronidase Deficiencies[Title/Abstract])) OR (Hurler-Scheie Syndrome[Title/Abstract])) OR (Hurler Scheie Syndrome[Title/Abstract])) OR (Mucopolysaccharidosis Type Ih S[Title/Abstract])	1930

#2	((((((((((((((((((Mucopolysaccharidosis 2[Title/Abstract]) OR (Hunter Syndrome[Title/Abstract])) OR (Syndrome, Hunter[Title/Abstract])) OR (Hunter Syndrome Gargoylism[Title/Abstract])) OR (Mucopolysaccharidosis Type 2[Title/Abstract])) OR (Mucopolysaccharidosis Type II[Title/Abstract])) OR (Hunter's Syndrome[Title/Abstract])) OR (Hunters Syndrome[Title/Abstract])) OR (Syndrome, Hunter's[Title/Abstract])) OR (Gargoylism, Hunter Syndrome[Title/Abstract])) OR (Sulfoiduronate Sulfatase Deficiency[Title/Abstract])) OR (Deficiency, Sulfoiduronate Sulfatase[Title/Abstract])) OR (Iduronate 2-Sulfatase Deficiency[Title/Abstract])) OR (Deficiency, Iduronate 2-Sulfatase[Title/Abstract])) OR (Iduronate 2 Sulfatase Deficiency[Title/Abstract])) OR (I2S Deficiency[Title/Abstract])) OR (Deficiency, I2S[Title/Abstract])) OR (Iduronate Sulfatase Deficiency[Title/Abstract])) OR (Deficiency, Iduronate Sulfatase[Title/Abstract])	1167
#3	((Mucopolysaccharidosis IIIs[Title/Abstract]) OR (Sanfilippo Syndrome[Title/Abstract])) OR (Sanfilippo Syndromes[Title/Abstract])) OR (Syndrome, Sanfilippo[Title/Abstract])) OR (Syndromes, Sanfilippo[Title/Abstract])) OR (Mucopolysaccharidosis 3[Title/Abstract])) OR (San Filippo's Syndrome[Title/Abstract])) OR (San Filippo Syndrome[Title/Abstract])) OR (San Filippos Syndrome[Title/Abstract])) OR (Syndrome, San Filippo's[Title/Abstract])) OR (Polydystrophic Oligophrenia[Title/Abstract])) OR (Oligophrenia, Polydystrophic[Title/Abstract])) OR (Oligophrenias, Polydystrophic[Title/Abstract])) OR (Polydystrophic Oligophrenias[Title/Abstract])) OR (Sanfilippo's Syndrome[Title/Abstract])) OR (Sanfilippos Syndrome[Title/Abstract])) OR (Syndrome, Sanfilippo's[Title/Abstract])) OR (MPS III D[Title/Abstract])) OR (Mucopolysaccharidosis Type 3	1947

	D[Title/Abstract])) OR (Mucopolysaccharidosis Type IIID[Title/Abstract])) OR (Mucopolysaccharidosis Type IIIDs[Title/Abstract])) OR (Sanfilippo Syndrome D[Title/Abstract])) OR (N-Acetylglucosamine-6-Sulfatase Deficiency[Title/Abstract])) OR (Deficiencies, N-Acetylglucosamine-6-Sulfatase[Title/Abstract])) OR (Deficiency, N-Acetylglucosamine-6-Sulfatase[Title/Abstract])) OR (N Acetylglucosamine 6 Sulfatase Deficiency[Title/Abstract])) OR (N-Acetylglucosamine-6-Sulfatase Deficiencies[Title/Abstract])) OR (MPS IIID[Title/Abstract])) OR (MPS IIIDs[Title/Abstract])) OR (MPS 3 D[Title/Abstract])) OR (N-Acetylglucosamine-6-Sulfate Sulfatase Deficiency[Title/Abstract])) OR (Deficiencies, N-Acetylglucosamine-6-Sulfate Sulfatase[Title/Abstract])) OR (Deficiency, N-Acetylglucosamine-6-Sulfate Sulfatase[Title/Abstract])) OR (N Acetylglucosamine 6 Sulfate Sulfatase Deficiency[Title/Abstract])) OR (N-Acetylglucosamine-6-Sulfate Sulfatase Deficiencies[Title/Abstract])) OR (Sulfatase Deficiencies, N-Acetylglucosamine-6-Sulfate[Title/Abstract])) OR (Sulfatase Deficiency, N-Acetylglucosamine-6-Sulfate[Title/Abstract])) OR (MPS III C[Title/Abstract])) OR (Acetyl-CoA:alpha-Glucosaminide N-Acetyltransferase Deficiency[Title/Abstract])) OR (Acetyl CoA:alpha Glucosaminide N Acetyltransferase Deficiency[Title/Abstract])) OR (Acetyl-CoA:alpha-Glucosaminide N-Acetyltransferase Deficiencies[Title/Abstract])) OR (Deficiencies, Acetyl-CoA:alpha-Glucosaminide N-Acetyltransferase[Title/Abstract])) OR (Deficiency, Acetyl-CoA:alpha-Glucosaminide N-Acetyltransferase[Title/Abstract])) OR (N-Acetyltransferase Deficiencies, Acetyl-CoA:alpha-Glucosaminide[Title/Abstract])) OR (N-Acetyltransferase Deficiency, Acetyl-CoA:alpha-Glucosaminide[Title/Abstract])) OR (Mucopolysaccharidosis Type IIIC[Title/Abstract])) OR	

	(Mucopolysaccharidosis Type IIICs[Title/Abstract])) OR (MPS 3 C[Title/Abstract])) OR (MPS3C[Title/Abstract])) OR (MPS IIIC[Title/Abstract])) OR (Mucopolysaccharidosis Type 3 C[Title/Abstract])) OR (Sanfilippo Syndrome C[Title/Abstract])) OR (MPS III A[Title/Abstract])) OR (MPS 3 A[Title/Abstract])) OR (Mucopolysaccharidosis Type 3 A[Title/Abstract])) OR (Mucopolysaccharidosis Type IIIA[Title/Abstract])) OR (Mucopolysaccharidosis Type IIIAs[Title/Abstract])) OR (Sanfilippo Syndrome A[Title/Abstract])) OR (Mucopolysaccharidosis Type 3 A Sanfilippo Syndrome[Title/Abstract])) OR (Sulfamidase Deficiency[Title/Abstract])) OR (Deficiencies, Sulfamidase[Title/Abstract])) OR (Deficiency, Sulfamidase[Title/Abstract])) OR (Sulfamidase Deficiencies[Title/Abstract])) OR (MPS IIIA[Title/Abstract])) OR (MPS3A[Title/Abstract])) OR (Heparan Sulfate Sulfatase Deficiency[Title/Abstract])) OR (MPS III B[Title/Abstract])) OR (Sanfilippo Syndrome B[Title/Abstract])) OR (MPS3B[Title/Abstract])) OR (Mucopolysaccharidosis Type IIIB[Title/Abstract])) OR (Mucopolysaccharidosis Type IIIBs[Title/Abstract])) OR (NAGLU Deficiency[Title/Abstract])) OR (Deficiencies, NAGLU[Title/Abstract])) OR (Deficiency, NAGLU[Title/Abstract])) OR (NAGLU Deficiencies[Title/Abstract])) OR (N-Acetyl-alpha-D-Glucosaminidase Deficiency[Title/Abstract])) OR (Deficiencies, N-Acetyl-alpha-D-Glucosaminidase[Title/Abstract])) OR (Deficiency, N-Acetyl-alpha-D-Glucosaminidase[Title/Abstract])) OR (N Acetyl alpha D Glucosaminidase Deficiency[Title/Abstract])) OR (N-Acetyl-alpha-D-Glucosaminidase Deficiencies[Title/Abstract])) OR (MPS IIIB[Title/Abstract])) OR (Mucopolysaccharidosis Type 3 B[Title/Abstract])) OR (MPS 3 B[Title/Abstract])) OR (Eccentroosteochondrodysplasia[Title/Abstract])) OR (Eccentroosteochondrodysplasias[Title/Abstract])) OR (Morquio Disease[Title/Abstract])) OR (Disease,	

	Morquio[Title/Abstract])) OR (Mucopolysaccharidosis Type IV[Title/Abstract])) OR (IV, Mucopolysaccharidosis Type[Title/Abstract])) OR (IVs, Mucopolysaccharidosis Type[Title/Abstract])) OR (Mucopolysaccharidosis Type IVs[Title/Abstract])) OR (Type IV, Mucopolysaccharidosis[Title/Abstract])) OR (Type IVs, Mucopolysaccharidosis[Title/Abstract])) OR (Morquio's Disease[Title/Abstract])) OR (Disease, Morquio's[Title/Abstract])) OR (Morquios Disease[Title/Abstract])) OR (Morquio's Syndrome[Title/Abstract])) OR (Morquios Syndrome[Title/Abstract])) OR (Syndrome, Morquio's[Title/Abstract])) OR (Mucopolysaccharidosis 4[Title/Abstract])) OR (Eccentro-Osteochondrodysplasia[Title/Abstract])) OR (Eccentro Osteochondrodysplasia[Title/Abstract])) OR (Eccentro-Osteochondrodysplasias[Title/Abstract])) OR (Morquio Syndrome[Title/Abstract])) OR (Morquio Syndromes[Title/Abstract])) OR (Syndrome, Morquio[Title/Abstract])) OR (Syndromes, Morquio[Title/Abstract])) OR (Mucopolysaccharidosis Type IV A[Title/Abstract])) OR (Morquio A Disease[Title/Abstract])) OR (Disease, Morquio A[Title/Abstract])) OR (Mucopolysaccharidosis Type IVA[Title/Abstract])) OR (MPS IV A[Title/Abstract])) OR (MPS IVA[Title/Abstract])) OR (Galactosamine-6-Sulfatase Deficiency[Title/Abstract])) OR (Deficiencies, Galactosamine-6-Sulfatase[Title/Abstract])) OR (Deficiency, Galactosamine-6-Sulfatase[Title/Abstract])) OR (Galactosamine 6 Sulfatase Deficiency[Title/Abstract])) OR (Galactosamine-6-Sulfatase Deficiencies[Title/Abstract])) OR (Morquio Syndrome A[Title/Abstract])) OR (Syndrome A, Morquio[Title/Abstract])) OR (Mucopolysaccharidosis Type IV B[Title/Abstract])) OR (Morquio Syndrome B[Title/Abstract])) OR (Morquio Syndrome, Type B[Title/Abstract])) OR (Mucopolysaccharidosis Type IVB[Title/Abstract])) OR	

	(Mucopolysaccharidosis Type IVBs[Title/Abstract])) OR (Morquio-B Disease[Title/Abstract])) OR (Disease, Morquio-B[Title/Abstract])) OR (Morquio B Disease[Title/Abstract])) OR (MPS IV B[Title/Abstract])) OR (MPS IVB[Title/Abstract])) OR (Mucopolysaccharidosis 4B[Title/Abstract])) OR (Mucopolysaccharidosis 4Bs[Title/Abstract])) OR (Morquio B Syndrome[Title/Abstract])) OR (Syndrome, Morquio B[Title/Abstract])) OR (Morquio's Disease Type B[Title/Abstract])) OR (GALNS Deficiency[Title/Abstract])) OR (Deficiencies, GALNS[Title/Abstract])) OR (Deficiency, GALNS[Title/Abstract])) OR (GALNS Deficiencies[Title/Abstract])	
#4	(((((((((((((((((((Arylsulfatase B Deficiency[Title/Abstract]) OR (Arylsulfatase B Deficiencies[Title/Abstract])) OR (Deficiencies, Arylsulfatase B[Title/Abstract])) OR (Deficiency, Arylsulfatase B[Title/Abstract])) OR (Maroteaux-Lamy Syndrome[Title/Abstract])) OR (Maroteaux Lamy Syndrome[Title/Abstract])) OR (Syndrome, Maroteaux-Lamy[Title/Abstract])) OR (Mucopolysaccharidosis Type VI[Title/Abstract])) OR (Type VI, Mucopolysaccharidosis[Title/Abstract])) OR (Mucopolysaccharidosis Type 6[Title/Abstract])) OR (Type 6, Mucopolysaccharidosis[Title/Abstract])) OR (N-Acetylgalactosamine-4-Sulfatase Deficiency[Title/Abstract])) OR (Deficiencies, N-Acetylgalactosamine-4-Sulfatase[Title/Abstract])) OR (Deficiency, N-Acetylgalactosamine-4-Sulfatase[Title/Abstract])) OR (N-Acetylgalactosamine-4-Sulfatase Deficiencies[Title/Abstract])) OR (Polydystrophic Dwarfism[Title/Abstract])) OR (Dwarfism, Polydystrophic[Title/Abstract])) OR (ARSB Deficiency[Title/Abstract])) OR (ARSB Deficiencies[Title/Abstract])) OR (Deficiencies, ARSB[Title/Abstract])) OR (Deficiency, ARSB[Title/Abstract])) OR	523

	(Mucopolysaccharidosis 6[Title/Abstract])	
#5	((((((((((((((((((Mucopolysaccharidosis VIIs[Title/Abstract]) OR (VIIs, Mucopolysaccharidosis[Title/Abstract])) OR (Sly Disease[Title/Abstract])) OR (Disease, Sly[Title/Abstract])) OR (beta-Glucuronidase Deficiency[Title/Abstract])) OR (Deficiencies, beta-Glucuronidase[Title/Abstract])) OR (Deficiency, beta-Glucuronidase[Title/Abstract])) OR (beta Glucuronidase Deficiency[Title/Abstract])) OR (beta-Glucuronidase Deficiencies[Title/Abstract])) OR (GUSB Deficiency[Title/Abstract])) OR (Deficiencies, GUSB[Title/Abstract])) OR (Deficiency, GUSB[Title/Abstract])) OR (GUSB Deficiencies[Title/Abstract])) OR (Mucopolysaccharidosis Type VII[Title/Abstract])) OR (Mucopolysaccharidosis Type VIIs[Title/Abstract])) OR (Type VII, Mucopolysaccharidosis[Title/Abstract])) OR (Type VIIs, Mucopolysaccharidosis[Title/Abstract])) OR (Mucopolysaccharidosis 7[Title/Abstract])) OR (Sly Syndrome[Title/Abstract])) OR (Syndrome, Sly[Title/Abstract])	1509
#6	(((((Mucopolysaccharidosis I[MeSH Terms]) OR (Mucopolysaccharidosis II[MeSH Terms])) OR (Mucopolysaccharidosis III[MeSH Terms])) OR (Mucopolysaccharidosis IV[MeSH Terms])) OR (Mucopolysaccharidosis VI[MeSH Terms])) OR (Mucopolysaccharidosis VII[MeSH Terms])	5308
#7	#1 OR #2 OR #3 OR #4 OR #5 OR #6	7473
#	((((((((((((((((randomized controlled study) OR (randomized controlled trial)) OR (randomized study)) OR (randomized trial))	159208

8	OR (randomized placebo-controlled study)) OR (randomized placebo-controlled trial)) OR (randomized placebo controlled)) OR (randomized placebo-controlled)) OR (randomized double-blind)) OR (randomized double blind)) OR (randomized)) OR (double-blind)) OR (randomized)) OR (placebo-controlled)) OR (Randomized Controlled Trials as Topic[MeSH Terms])) OR (Randomized Controlled Trial[Publication Type])) OR (Controlled Clinical Trial[Publication Type])	3
#9	#7 AND #8	132

Database: Web of Science<inception to Aug 22, 2023>

(648)

TS=(Mucopolysaccharidosis Is OR Mucopolysaccharidosis Type I OR Lipochondrodystrophy OR Lipochondrodystrophies OR Hurler Syndrome OR Hurler's Disease OR Hurler's Syndrome OR Gargoylism OR Gargoylisms OR Hurler Syndrome Gargoylism OR Mucopolysaccharidosis Type Ih OR Mucopolysaccharidosis Type Ihs OR Hurler Disease OR Pfaundler-Hurler Syndrome OR Scheie Syndrome OR Scheie's Syndrome OR Mucopolysaccharidosis Type Is OR Mucopolysaccharidosis I-S OR Mucopolysaccharidosis I S OR Mucopolysaccharidosis V OR Mucopolysaccharidosis 5 OR alpha-L-Iduronidase Deficiency OR alpha L Iduronidase Deficiency OR alpha-L-Iduronidase Deficiencies OR Hurler-Scheie Syndrome OR Hurler Scheie Syndrome OR Mucopolysaccharidosis Type Ih S OR Mucopolysaccharidosis 2 OR Hunter Syndrome OR Mucopolysaccharidosis Type 2 OR Mucopolysaccharidosis Type II OR Hunter's Syndrome OR Hunters Syndrome OR Sulfoiduronate Sulfatase Deficiency OR Iduronate 2-Sulfatase Deficiency OR Iduronate 2 Sulfatase Deficiency OR

I2S Deficiency OR Iduronate Sulfatase Deficiency OR Mucopolysaccharidosis IIIs OR Sanfilippo Syndrome OR Sanfilippo Syndromes OR Mucopolysaccharidosis 3 OR San Filippo's Syndrome OR San Filippo Syndrome OR San Filippos Syndrome OR Polydystrophic Oligophrenia OR Polydystrophic Oligophrenias OR Sanfilippo's Syndrome OR Sanfilippos Syndrome OR MPS III D OR Mucopolysaccharidosis Type 3 D OR Mucopolysaccharidosis Type IIID OR Mucopolysaccharidosis Type IIIDs OR Sanfilippo Syndrome D OR N-Acetylglucosamine-6-Sulfatase Deficiency OR N Acetylglucosamine 6 Sulfatase Deficiency OR N-Acetylglucosamine-6-Sulfatase Deficiencies OR MPS IIID OR MPS IIIDs OR MPS 3 D OR N-Acetylglucosamine-6-Sulfate Sulfatase Deficiency OR N Acetylglucosamine 6 Sulfate Sulfatase Deficiency OR N-Acetylglucosamine-6-Sulfate Sulfatase Deficiencies OR MPS III C OR Mucopolysaccharidosis Type IIIC OR Mucopolysaccharidosis Type IIICs OR MPS 3 C OR MPS3C OR MPS IIIC OR Mucopolysaccharidosis Type 3 C OR Sanfilippo Syndrome C OR MPS III A OR MPS 3 A OR Mucopolysaccharidosis Type 3 A OR Mucopolysaccharidosis Type IIIA OR Mucopolysaccharidosis Type IIIAs OR Sanfilippo Syndrome A OR Mucopolysaccharidosis Type 3 A Sanfilippo Syndrome OR Sulfamidase Deficiency OR Sulfamidase Deficiencies OR MPS IIIA OR MPS3A OR Heparan Sulfate Sulfatase Deficiency OR MPS III B OR Sanfilippo Syndrome B OR MPS3B OR Mucopolysaccharidosis Type IIIB OR Mucopolysaccharidosis Type IIIBs OR NAGLU Deficiency OR NAGLU Deficiencies OR N-Acetyl-alpha-D-Glucosaminidase Deficiency OR N Acetyl alpha D Glucosaminidase Deficiency OR N-Acetyl-alpha-D-Glucosaminidase Deficiencies OR MPS IIIB OR Mucopolysaccharidosis Type 3 B OR MPS 3 B OR Eccentroosteochondrodysplasia OR Eccentroosteochondrodysplasias OR Morquio Disease OR Mucopolysaccharidosis Type IVs OR Morquio's Disease OR Morquios Disease OR Morquio's Syndrome OR Morquios Syndrome OR Mucopolysaccharidosis 4 OR Eccentro-Osteochondrodysplasia OR Eccentro Osteochondrodysplasia OR Eccentro-Osteochondrodysplasias OR Morquio Syndrome OR Morquio Syndromes OR Mucopolysaccharidosis Type IV A OR Morquio A Disease OR Mucopolysaccharidosis Type IVA OR MPS IV A OR MPS IVA OR

Galactosamine-6-Sulfatase Deficiency OR Galactosamine 6 Sulfatase Deficiency OR Galactosamine-6-Sulfatase Deficiencies OR Morquio Syndrome A OR Mucopolysaccharidosis Type IV B OR Morquio Syndrome B OR Mucopolysaccharidosis Type IVB OR Mucopolysaccharidosis Type IVBs OR Morquio-B Disease OR Morquio B Disease OR MPS IV B OR MPS IVB OR Mucopolysaccharidosis 4B OR Mucopolysaccharidosis 4Bs OR Morquio B Syndrome OR Morquio's Disease Type B OR GALNS Deficiency OR GALNS Deficiencies OR Arylsulfatase B Deficiency OR Arylsulfatase B Deficiencies OR Maroteaux-Lamy Syndrome OR Maroteaux Lamy Syndrome OR Mucopolysaccharidosis Type VI OR Mucopolysaccharidosis Type 6 OR N-Acetylgalactosamine-4-Sulfatase Deficiency OR N-Acetylgalactosamine-4-Sulfatase Deficiencies OR Polydystrophic Dwarfism OR ARSB Deficiency OR ARSB Deficiencies OR Mucopolysaccharidosis 6 OR Mucopolysaccharidosis VIIs OR Sly Disease OR beta-Glucuronidase Deficienc OR beta Glucuronidase Deficiency OR beta-Glucuronidase Deficiencies OR GUSB Deficiency OR GUSB Deficiencies OR Mucopolysaccharidosis Type VII OR Mucopolysaccharidosis Type VIIs OR Mucopolysaccharidosis 7 OR Sly Syndrome OR Mucopolysaccharidosis I OR Mucopolysaccharidosis II OR Mucopolysaccharidosis III OR Mucopolysaccharidosis IV OR Mucopolysaccharidosis VI OR Mucopolysaccharidosis VII) AND TS=(randomized controlled study OR randomized controlled trial OR randomized study OR randomized trial OR randomized placebo-controlled study OR randomized placebo-controlled trial OR randomized placebo controlled OR randomized placebo-controlled OR randomized double-blind OR randomized double blind OR randomized OR double-blind OR randomized OR placebo-controlled OR Randomized Controlled Trials as Topic OR Randomized Controlled Trial OR Controlled Clinical Trial)

Database: Cochrane library<inception to Aug 22, 2023>

#1	Mucopolysaccharidosis	90
#2	Mucopolysaccharidosis Is or Mucopolysaccharidosis Type I or Lipochondrodystrophy or Lipochondrodystrophies or Hurler Syndrome or Hurler's Disease or Disease, Hurler's or Hurler's Syndrome or Syndrome, Hurler's or Gargoylism or Gargoylisms or Gargoylism, Hurler Syndrome or Hurler Syndrome Gargoylism or Mucopolysaccharidosis Type Ih or Mucopolysaccharidosis Type Ihs or Type Ih, Mucopolysaccharidosis or Type Ihs, Mucopolysaccharidosis or Hurler Disease or Pfaundler-Hurler Syndrome or Scheie Syndrome or Scheie's Syndrome or Syndrome, Scheie's or Mucopolysaccharidosis Type Is or Mucopolysaccharidosis I-S or Mucopolysaccharidosis I S or Mucopolysaccharidosis V or Mucopolysaccharidosis 5 or alpha-L-Iduronidase Deficiency or alpha L Iduronidase Deficiency or alpha-L-Iduronidase Deficiencies or Hurler-Scheie Syndrome or Hurler Scheie Syndrome or Mucopolysaccharidosis Type Ih S	167
#3	Mucopolysaccharidosis 2 or Hunter Syndrome or Syndrome, Hunter or Hunter Syndrome Gargoylism or Mucopolysaccharidosis Type 2 or Mucopolysaccharidosis Type II or Hunter's Syndrome or Hunters Syndrome or Syndrome, Hunter's or Gargoylism, Hunter Syndrome or Sulfoiduronate Sulfatase Deficiency or Deficiency, Sulfoiduronate Sulfatase or Iduronate 2 Sulfatase Deficiency or I2S Deficiency or Deficiency, I2S or Iduronate Sulfatase Deficiency or Deficiency, Iduronate Sulfatase	189
#4	Mucopolysaccharidosis IIIs or Sanfilippo Syndrome or Sanfilippo Syndromes or Syndrome, Sanfilippo or Syndromes, Sanfilippo or Mucopolysaccharidosis 3 or San Filippo's Syndrome or San Filippo Syndrome or San Filippos Syndrome or Syndrome, San	888

	Filippo's or Polydystrophic Oligophrenia or Oligophrenia, Polydystrophic or Oligophrenias, Polydystrophic or Polydystrophic Oligophrenias or Sanfilippo's Syndrome or Sanfilippos Syndrome or Syndrome, Sanfilippo's or MPS III D or Mucopolysaccharidosis Type 3 D or Mucopolysaccharidosis Type IIID or Mucopolysaccharidosis Type IIIDs or Sanfilippo Syndrome D or N Acetylglucosamine 6 Sulfatase Deficiency or MPS IIID or MPS IIIDs or MPS 3 D or N Acetylglucosamine 6 Sulfate Sulfatase Deficiency or MPS III C or Mucopolysaccharidosis Type IIIC or Mucopolysaccharidosis Type IIICs or MPS 3 C or MPS3C or MPS IIIC or Mucopolysaccharidosis Type 3 C or Sanfilippo Syndrome C or MPS III A or MPS 3 A or Mucopolysaccharidosis Type 3 A or Mucopolysaccharidosis Type IIIA or Mucopolysaccharidosis Type IIIAs or Sanfilippo Syndrome A or Mucopolysaccharidosis Type 3 A Sanfilippo Syndrome or Sulfamidase Deficiency or Deficiencies, Sulfamidase or Deficiency, Sulfamidase or Sulfamidase Deficiencies or MPS IIIA or MPS3A or Heparan Sulfate Sulfatase Deficiency or MPS III B or Sanfilippo Syndrome B or MPS3B or Mucopolysaccharidosis Type IIIB or Mucopolysaccharidosis Type IIIBs or NAGLU Deficiency or Deficiencies, NAGLU or Deficiency, NAGLU or NAGLU Deficiencies or Mucopolysaccharidosis Type 3 B or MPS 3 B or Eccentroosteochondrodysplasia or Eccentroosteochondrodysplasias or Morquio Disease or Disease, Morquio or Mucopolysaccharidosis Type IV or IV, Mucopolysaccharidosis Type or IVs, Mucopolysaccharidosis Type or Mucopolysaccharidosis Type IVs or Type IV, Mucopolysaccharidosis or Type IVs, Mucopolysaccharidosis or Morquio's Disease or Disease, Morquio's or Morquios Disease or Morquio's Syndrome or Morquios Syndrome or Syndrome, Morquio's or Mucopolysaccharidosis 4 or Eccentro Osteochondrodysplasia or Morquio Syndrome or Morquio Syndromes or Syndrome,	

	Morquio or Syndromes, Morquio or Mucopolysaccharidosis Type IV A or Morquio A Disease or Disease, Morquio A or Mucopolysaccharidosis Type IVA or MPS IV A or MPS IVA or Galactosamine 6 Sulfatase Deficiency or Morquio Syndrome A or Syndrome A, Morquio or Mucopolysaccharidosis Type IV B or Morquio Syndrome B or Morquio Syndrome, Type B or Mucopolysaccharidosis Type IVB or Mucopolysaccharidosis Type IVBs or Morquio B Disease or MPS IV B or MPS IVB or Mucopolysaccharidosis 4B or Mucopolysaccharidosis 4Bs or Morquio B Syndrome or Syndrome, Morquio B or Morquio's Disease Type B or GALNS Deficiency or Deficiencies, GALNS or Deficiency, GALNS or GALNS Deficiencies	
#5	Arylsulfatase B Deficiency or Arylsulfatase B Deficiencies or Deficiencies, Arylsulfatase B or Deficiency, Arylsulfatase B or Maroteaux-Lamy Syndrome or Maroteaux Lamy Syndrome or Mucopolysaccharidosis Type VI or Type VI, Mucopolysaccharidosis or Mucopolysaccharidosis Type 6 or Type 6, Mucopolysaccharidosis or Polydystrophic Dwarfism or Dwarfism, Polydystrophic or ARSB Deficiency or ARSB Deficiencies or Deficiencies, ARSB or Deficiency, ARSB or Mucopolysaccharidosis 6	108
#6	Mucopolysaccharidosis VIIs or VIIs, Mucopolysaccharidosis or Sly Disease or Disease, Sly or beta Glucuronidase Deficiency or GUSB Deficiency or Deficiencies, GUSB or Deficiency, GUSB or GUSB Deficiencies or Mucopolysaccharidosis Type VII or Mucopolysaccharidosis Type VIIs or Type VII, Mucopolysaccharidosis or Type VIIs, Mucopolysaccharidosis or Mucopolysaccharidosis 7 or Sly Syndrome or Syndrome, Sly	73
#7	#1 or#2 or#3 or#4 or#5 or#6	979

#8	randomized controlled study or randomized controlled trial or randomized study or randomized trial or randomized placebo-controlled study or randomized placebo-controlled trial or randomized placebo controlled or randomized placebo-controlled or randomized double-blind or randomized double blind or randomized or double-blind or randomized or placebo-controlled or Randomized Controlled Trials as Topic or Randomized Controlled Trial or Controlled Clinical Trial	1225 773
9#	#7 and #8	719

Database: Embase<inception to Aug 22, 2023>

#1	Mucopolysaccharidosis Is OR Mucopolysaccharidosis Type I OR Lipochondrodystrophy OR Lipochondrodystrophies OR Hurler Syndrome OR Hurlers Disease OR Disease, Hurlers OR Hurlers Syndrome OR Syndrome, Hurlers OR Gargoylism OR Gargoylisms OR Gargoylism, Hurler Syndrome OR Hurler Syndrome Gargoylism OR Mucopolysaccharidosis Type Ih OR Mucopolysaccharidosis Type Ihs OR Type Ih, Mucopolysaccharidosis OR Type Ihs, Mucopolysaccharidosis OR Hurler Disease OR Pfaundler-Hurler Syndrome OR Scheie Syndrome OR Scheies Syndrome OR Syndrome, Scheies OR Mucopolysaccharidosis Type Is OR Mucopolysaccharidosis I-S OR Mucopolysaccharidosis I S OR Mucopolysaccharidosis V OR Mucopolysaccharidosis 5 OR alpha-L-Iduronidase Deficiency OR alpha L Iduronidase Deficiency OR alpha-L-Iduronidase Deficiencies OR Hurler-Scheie Syndrome OR Hurler Scheie Syndrome OR Mucopolysaccharidosis Type Ih S	148
#2	Mucopolysaccharidosis 2 OR Hunter Syndrome OR Syndrome, Hunter OR Hunter Syndrome Gargoylism OR Mucopolysaccharidosis Type 2 OR Mucopolysaccharidosis Type II OR Hunters Syndrome OR Hunters Syndrome OR Syndrome, Hunters OR Gargoylism, Hunter Syndrome OR Sulfoiduronate Sulfatase Deficiency OR Deficiency, Sulfoiduronate Sulfatase OR Iduronate 2-Sulfatase Deficiency OR Deficiency, Iduronate 2-Sulfatase OR Iduronate 2 Sulfatase Deficiency OR I2S Deficiency OR Deficiency, I2S OR Iduronate Sulfatase Deficiency OR Deficiency, Iduronate Sulfatase	618
#3	Arylsulfatase B Deficiency OR Arylsulfatase B Deficiencies OR Deficiencies, Arylsulfatase B OR Deficiency, Arylsulfatase B OR Maroteaux-Lamy Syndrome OR Maroteaux Lamy Syndrome OR Syndrome, Maroteaux-Lamy OR Mucopolysaccharidosis	10791

	Type VI OR Type VI, Mucopolysaccharidosis OR Mucopolysaccharidosis Type 6 OR Type 6, Mucopolysaccharidosis OR N-Acetylgalactosamine-4-Sulfatase Deficiency OR Deficiencies, N-Acetylgalactosamine-4-Sulfatase OR Deficiency, N-Acetylgalactosamine-4-Sulfatase OR N-Acetylgalactosamine-4-Sulfatase Deficiencies OR Polydystrophic Dwarfism OR Dwarfism, Polydystrophic OR ARSB Deficiency OR ARSB Deficiencies OR Deficiencies, ARSB OR Deficiency, ARSB OR Mucopolysaccharidosis 6	
#4	Mucopolysaccharidosis VIIs OR VIIs, Mucopolysaccharidosis OR Sly Disease OR Disease, Sly OR beta-Glucuronidase Deficiency OR Deficiencies, beta-Glucuronidase OR Deficiency, beta-Glucuronidase OR beta Glucuronidase Deficiency OR beta-Glucuronidase Deficiencies OR GUSB Deficiency OR Deficiencies, GUSB OR Deficiency, GUSB OR GUSB Deficiencies OR Mucopolysaccharidosis Type VII OR Mucopolysaccharidosis Type VIIs OR Type VII, Mucopolysaccharidosis OR Type VIIs, Mucopolysaccharidosis OR Mucopolysaccharidosis 7 OR Sly Syndrome OR Syndrome, Sly	11740
#5	Mucopolysaccharidosis IIIs OR Sanfilippo Syndrome OR Sanfilippo Syndromes OR Syndrome, Sanfilippo OR Syndromes, Sanfilippo OR Mucopolysaccharidosis 3 OR San Filippos Syndrome OR San Filippo Syndrome OR San Filippos Syndrome OR Syndrome, San Filippos OR Polydystrophic Oligophrenia OR Oligophrenia, Polydystrophic OR Oligophrenias, Polydystrophic OR Polydystrophic Oligophrenias OR Sanfilippos Syndrome OR Sanfilippos Syndrome OR Syndrome, Sanfilippos OR MPS III D OR Mucopolysaccharidosis Type 3 D OR Mucopolysaccharidosis Type IIID OR Mucopolysaccharidosis Type IIIDs OR Sanfilippo Syndrome D	16270

#6	N- N-Acetylglucosamine-6-Sulfatase Deficiency OR Deficiencies, N-Acetylglucosamine-6-Sulfatase OR Deficiency, N-Acetylglucosamine-6-Sulfatase OR N Acetylglucosamine 6 Sulfatase Deficiency OR N-Acetylglucosamine-6-Sulfatase Deficiencies OR MPS IIID OR MPS IIIDs OR MPS 3 D OR N-Acetylglucosamine-6-Sulfate Sulfatase Deficiency OR Deficiencies, N-Acetylglucosamine-6-Sulfate Sulfatase OR Deficiency, N-Acetylglucosamine-6-Sulfate Sulfatase OR N Acetylglucosamine 6 Sulfate Sulfatase Deficiency OR N-Acetylglucosamine-6-Sulfate Sulfatase Deficiencies OR Sulfatase Deficiencies, N-Acetylglucosamine-6-Sulfate OR Sulfatase Deficiency, N-Acetylglucosamine-6-Sulfate OR MPS III C	9250
#7	Mucopolysaccharidosis Type IIIC OR Mucopolysaccharidosis Type IIICs OR MPS 3 C OR MPS3C OR MPS IIIC OR Mucopolysaccharidosis Type 3 C OR Sanfilippo Syndrome C OR MPS III A OR MPS 3 A OR Mucopolysaccharidosis Type 3 A OR Mucopolysaccharidosis Type IIIA OR Mucopolysaccharidosis Type IIIAs OR Sanfilippo Syndrome A OR Mucopolysaccharidosis Type 3 A Sanfilippo Syndrome OR Sulfamidase Deficiency OR Deficiencies, Sulfamidase OR Deficiency, Sulfamidase OR Sulfamidase Deficiencies OR MPS IIIA OR MPS3A OR Heparan Sulfate Sulfatase Deficiency OR MPS III B OR Sanfilippo Syndrome B OR MPS3B OR Mucopolysaccharidosis Type IIIB OR Mucopolysaccharidosis Type IIIBs OR NAGLU Deficiency OR Deficiencies, NAGLU OR Deficiency, NAGLU OR NAGLU Deficiencies OR N-Acetyl-alpha-D-Glucosaminidase Deficiency OR Deficiencies, N-Acetyl-alpha-D-Glucosaminidase OR Deficiency, N-Acetyl-alpha-D-Glucosaminidase OR N Acetyl alpha D Glucosaminidase Deficiency OR N-Acetyl-alpha-D-Glucosaminidase Deficiencies OR MPS IIIB OR Mucopolysaccharidosis Type 3 B OR MPS 3 B OR Eccentroosteochondrodysplasia OR	34962

	Eccentroosteochondrodysplasias OR Morquio Disease OR Disease, Morquio OR Mucopolysaccharidosis Type IV OR IV, Mucopolysaccharidosis Type OR IVs, Mucopolysaccharidosis Type OR Mucopolysaccharidosis Type IVs OR Type IV, Mucopolysaccharidosis OR Type IVs, Mucopolysaccharidosis OR Morquios Disease OR Disease, Morquios OR Morquios Disease OR Morquios Syndrome OR Morquios Syndrome OR Syndrome, Morquios OR Mucopolysaccharidosis 4 OR Eccentro-Osteochondrodysplasia OR Eccentro Osteochondrodysplasia OR Eccentro-Osteochondrodysplasias OR Morquio Syndrome OR Morquio Syndromes OR Syndrome, Morquio OR Syndromes, Morquio OR Mucopolysaccharidosis Type IV A OR Morquio A Disease OR Disease, Morquio A OR Mucopolysaccharidosis Type IVA OR MPS IV A OR MPS IVA OR Galactosamine-6-Sulfatase Deficiency OR Deficiencies, Galactosamine-6-Sulfatase OR Deficiency, Galactosamine-6-Sulfatase OR Galactosamine 6 Sulfatase Deficiency OR Galactosamine-6-Sulfatase Deficiencies OR Morquio Syndrome A OR Syndrome A, Morquio OR Mucopolysaccharidosis Type IV B OR Morquio Syndrome B OR Morquio Syndrome, Type B OR Mucopolysaccharidosis Type IVB OR Mucopolysaccharidosis Type IVBs OR Morquio-B Disease OR Disease, Morquio-B OR Morquio B Disease OR MPS IV B OR MPS IVB OR Mucopolysaccharidosis 4B OR Mucopolysaccharidosis 4Bs OR Morquio B Syndrome OR Syndrome, Morquio B OR Morquios Disease Type B OR GALNS Deficiency OR Deficiencies, GALNS OR Deficiency, GALNS OR GALNS Deficiencies	
#8	'mucopolysaccharidosis'/exp	15621
#9	#1 OR #2 OR #3 OR #4 OR #5 OR #6 OR #7 OR #8	40715

#10	randomized controlled study or randomized controlled trial or randomized study or randomized trial or randomized placebo-controlled study or randomized placebo-controlled trial or randomized placebo controlled or randomized placebo-controlled or randomized double-blind or randomized double blind or randomized or double-blind or randomized or placebo-controlled or Randomized Controlled Trials as Topic or Randomized Controlled Trial or Controlled Clinical Trial	1933073
#11	#9 AND #10	2072
