# Supplementary material for: Efficacy of different treatment strategies in patients with mucopolysaccharidosis: a systematic review and network meta-analysis of randomized controlled trials
Source: Orphanet J Rare Dis. 2025 May 2;20:211. doi: 10.1186/s13023-025-03735-y (PMC12049060; doi:10.1186/s13023-025-03735-y)
Supplement: Supplementary file 3 — Supplementary Material 3: Appendix 3. Included studies. [file 13023_2025_3735_MOESM3_ESM.pdf]

Appendix 3 Included studies

Table 1. Study selection

No.	Type	Study (Author year)	Intervention	Dose	Age mean (y)	n	Duration (wks)	Blinding	Indications
1	I	Wraith 2004[1]	laronidase	0.58mg/kg_QW	15.6±9.0	22	26	2	6MWT、FVC
			placebo	NA	15.4±8.3	23			
2		Hennermann 2016[2]	pentosan polysulphate	1mg/kg_QW	35.6±6.4	2	24	NA	uGAG
			pentosan polysulphate	2mg/kg_QW	35.6±6.4	2			
3	II	Sohn 2013[3]	Idursulfase	0.5mg/kg_QW	11.7±9.0	10	24	1	6MWT、uGAG、FVC
			Idursulfase	1mg/kg_QW	11.5±4.7	10			
			placebo	0.5mg/kg_QW	10.8±4.6	11			
4		Muenzer 2022[4]	Idursulfase	10mg IT	5.0±1.5	32	52	1	uGAG
			placebo	NA	5.3±2.6	15			
5		Muenzer 2016[5]	Idursulfase	10mg IT	4.3±0.8	4	27	NA	CSF GAG
			Idursulfase	30mg IT	6.9±1.7	4			
			Idursulfase	1mg IT	5.6±1.8	4			
			placebo	NA	8.6±2.5	4			
6		Muenzer 2006[6]	Idursulfase	0.5mg/kg_Q2W	14.4±6.8	32	53	2	Liver volumes、uGAG、FVC、6MWT
			Idursulfase	0.5mg/kg_QW	15.1±6.3	32			
			placebo	NA	13.1±4.2	12			
7		Muenzer 2007[7]	Idursulfase	0.15mg/kg_Q2W	11.0±2.6	3	24	2	Liver volumes、uGAG
			Idursulfase	0.5mg/kg_Q2W	20±0	3			
			Idursulfase	1.5mg/kg_Q2W	8.0±2.0	3			
			placebo	NA	16.7±3.5	3			

No.	Type	Study (Author year)	Intervention	Dose	Age mean (y)	n	Duration (wks)	Blinding	Indications
7	II	Muenzer 2007[7]	Idursulfase	0.15mg/kg_Q2W	11.0±2.6	4	48	2	Liver volumes
			Idursulfase	0.5mg/kg_Q2W	20±0	4			
			Idursulfase	1.5mg/kg_Q2W	8±2.0	4			
8		Muenzer 2022[8]	Idursulfase	10mg IT	5.7±2.1	10	12	NA	CSF GAG
			Idursulfase	30mg IT	7.9±2.7	5			
9		Giugliani 2021[9]	pabinafusp alfa	1.0mg/kg	8.8±9.0	8	26	NA	Liver volumes
			pabinafusp alfa	2.0mg/kg	12.9±14.2	5			
			pabinafusp alfa	4.0mg/kg	19.6±18.8	6			
10	III	Jones 2016[10]	rhHNS	10mg_Q4W IT	9±4.6	4	22	NA	uGAG
			rhHNS	45mg_Q4W IT	9.25±9.9	4			
			rhHNS	90mg_Q4W IT	10.5±8.5	4			
11		Ghosh 2021[11]	Genistein	160 mg/kg/day	7.8±3.2	9	48	2	uGAG
			placebo	NA	7.9±4.8	11			
			Genistein	160 mg/kg/day	7.8±3.2	9	96		cognitive DQ score
			placebo	NA	7.9±4.8	11			
12		Wijburg 2019[12]	rhHNS	45mg_Q2W IT	2.5±0.8	7	48	NA	cognitive DQ score、uGAG
			rhHNS	45mg_Q4W IT	2.8±0.8	7			
			placebo	NA	2.7±0.8	7			
13		Ruijter 2012[13]	Genistein	10 mg/kg/day	17.1±12.6	15	24	2	uGAG
			placebo	NA	19.6±17.8	15			
14		Wijburg 2021[14]	rhHNS	45mg_Q2W IT	2.5±0.8	7	72	NA	cognitive DQ score
			rhHNS	45mg_Q4W IT	2.6±0.7	6			

No.	Type	Study (Author year)	Intervention	Dose	Age mean (y)	n	Duration (wks)	Blinding	Outcomes
14	III	Wijburg 2021[14]	rhHNS	45mg_Q2W IT	3.2±0.5	7	48	NA	cognitive DQ score
			rhHNS	45mg_Q4W IT	2.5±0.8	6			
			placebo	NA	2.6±0.7	4			
15	IV	Hendriksz 2014[15]	elosulfase_alfa	2.0mg/kg_Q2W	15.3±10.79	59	24	2	6MWT、3MSCT、uKS
			elosulfase_alfa	2.0mg/kg_QW	13.1±8.10	59			
			placebo	NA	15.0±11.30	58			
16		Berger 2017[16]	elosulfase_alfa	2.0mg/kg_QW	12.5±4.2	15	52	2	6MWT、3MSCT
			elosulfase_alfa	4.0mg/kg_QW	11.5±2.5	10			
			elosulfase_alfa	2.0mg/kg_QW	12.5±4.2	15	24		6MWT、3MSCT
			elosulfase_alfa	4.0mg/kg_QW	11.5±2.5	10			
17		Hughes 2017[17]	elosulfase_alfa	2.0mg/kg_QW	30.9±9.7	37	120	2	6MWT、3MSCT、uKS
			placebo	NA	26.9±7.8	10	2y		
18		Hendriksz 2016[18]	elosulfase_alfa	2.0mg/kg_QW	14.4±10.2	173	NA	2	6MWT、3MSCT、uKS
			placebo	NA	16.3±12.2	97			
19		Burton 2015[19]	elosulfase_alfa	2.0mg/kg_QW	14.9±9.32	15	27	2	6MWT、3MSCT、uKS
			elosulfase_alfa	4.0mg/kg_QW	12.0±3.16	10			
20	VI	Guffon 2022[20]	galsulfase+placebo	NA	20.4±6.1	5	26	2	6MWT、uGAG、FVC、MVV
			galsulfase+odiparcil	500 mg/day	30.0±19.3	5			
			galsulfase+odiparcil	1000 mg/day	24.0±6.1	5			
			odiparcil	1000 mg/day	29.4±8.4	5			

No.	Type	Study (Author year)	Intervention	Dose	Age mean (y)	n	Duration (wks)	Blinding	Indications
21	VI	Harmatz 2006[21]	rhASB	1.0mg/kg_QW	13.7±6.47	19	24	2	uGAG、FVC、MVV
			placebo	NA	10.7±4.35	20			
22		Harmatz 2004[22]	rhASB	1.0mg/kg	11.7±4.5	3	48	2	6MWT
			rhASB	0.2mg/kg	10.3±3.1	3			
23		Harmatz 2004[23]	galsulfase	1.0 mg/kg_QW	6.1±3.9	2	52	NA	uGAG
			galsulfase	2.0 mg/kg_QW	12.4±0.4	2			

6MWT: 6-minute walk test
3MSCT: 3-minute stair climb test (stairs/min)
uGAG: urine glycosaminoglycan
CSF GAG: urine glycosaminoglycan
uKS: urine keratan sulfate
MVV: Maximum voluntary ventilation
FVC: forced vital capacity
Cognitive DQ score: BSID-III cognitive DQ score

The primary endpoint was the extent of reduction in urinary GAG excretion. Secondary endpoints included liver and spleen size, 6-minute walk test (6MWT)

Reference list for included studies

[1] WRAITH J E, CLARKE L A, BECK M, et al. Enzyme replacement therapy for mucopolysaccharidosis I: A randomized, double-blinded, placebo-controlled, multinational study of recombinant human α-L-iduronidase (laronidase) [J]. Journal of Pediatrics, 2004, 144(5): 581-8.

[2] HENNERMANN J B, GöKCE S, SOLYOM A, et al. Treatment with pentosan polysulphate in patients with MPS I: results from an open label, randomized, monocentric phase II study [J]. Journal of Inherited Metabolic Disease, 2016, 39(6): 831-7.

[3] SOHN Y B, CHO S Y, PARK S W, et al. Phase I/II clinical trial of enzyme replacement therapy with idursulfase beta in patients with mucopolysaccharidosis II (Hunter Syndrome) [J]. Journal of Inherited Metabolic Disease, 2013, 36(2): S260.

[4] MUENZER J, BURTON B K, HARMATZ P, et al. Intrathecal idursulfase-IT in patients with neuronopathic mucopolysaccharidosis II: Results from a phase 2/3 randomized study [J]. Molecular Genetics and Metabolism, 2022, 137(1-2): 127-39.

[5] MUENZER J, HENDRIKSZ C J, FAN Z, et al. A phase I/II study of intrathecal idursulfase-IT in children with severe mucopolysaccharidosis II [J]. Genetics in Medicine, 2016, 18(1): 73-81.

[6] MUENZER J, WRAITH J E, BECK M, et al. A phase II/III clinical study of enzyme replacement therapy with idursulfase in mucopolysaccharidosis II (Hunter syndrome) [J]. Genetics in Medicine, 2006, 8(8): 465-73.

[7] MUENZER J, GUCSAVAS-CALIKOGLU M, MCCANDLESS S E, et al. A phase I/II clinical trial of enzyme replacement therapy in mucopolysaccharidosis II (Hunter syndrome) [J]. Molecular Genetics and Metabolism, 2007, 90(3 SPEC. ISS.): 329-37.

[8] MUENZER J, VIJAYARAGHAVAN S, STEIN M, et al. Long-term open-label phase I/II extension study of intrathecal idursulfase-IT in the treatment of neuronopathic mucopolysaccharidosis II [J]. Genetics in Medicine, 2022, 24(7): 1437-48.

[9] GIUGLIANI R, MARTINS A M, SO S, et al. Iduronate-2-sulfatase fused with anti-hTfR antibody, pabinafusp alfa, for MPS-II: A phase 2 trial in Brazil [J]. Molecular Therapy, 2021, 29(7): 2378-86.

[10] JONES S A, BREEN C, HEAP F, et al. A phase 1/2 study of intrathecal heparan-N-sulfatase in patients with mucopolysaccharidosis IIIA [J]. Mol Genet Metab, 2016, 118(3): 198-205.

[11] GHOSH A, RUST S, LANGFORD-SMITH K, et al. High dose genistein in Sanfilippo syndrome: A randomised controlled trial [J]. Journal of Inherited Metabolic Disease, 2021, 44(5): 1248-62.

[12] WIJBURG F A, WHITLEY C B, MUENZER J, et al. Intrathecal heparan-N-sulfatase in patients with Sanfilippo syndrome type A: A phase IIb randomized

trial [J]. Molecular Genetics and Metabolism, 2019, 126(2): 121-30.
[13] DE RUIJTER J, VALSTAR M J, NARAJCZYK M, et al. Genistein in Sanfilippo disease: A randomized controlled crossover trial [J]. Annals of Neurology, 2012, 71(1): 110-20.
[14] WIJBURG F A, WHITLEY C B, MUENZER J, et al. A multicenter open-label extension study of intrathecal heparan-N-sulfatase in patients with Sanfilippo syndrome type A [J]. Molecular Genetics and Metabolism, 2021, 134(1-2): 175-81.
[15] HENDRIKSZ C J, BURTON B, FLEMING T R, et al. Efficacy and safety of enzyme replacement therapy with BMN 110 (elosulfase alfa) for Morquio A syndrome (mucopolysaccharidosis IVA): a phase 3 randomised placebo-controlled study [J]. Journal of Inherited Metabolic Disease, 2014, 37(6): 979-90.
[16] BERGER K I, BURTON B K, LEWIS G D, et al. Cardiopulmonary Exercise Testing Reflects Improved Exercise Capacity in Response to Treatment in Morquio A Patients: Results of a 52-Week Pilot Study of Two Different Doses of Elosulfase Alfa [J]. JIMD reports, 2018, 42: 9-17.
[17] HUGHES D, GIUGLIANI R, GUFFON N, et al. Clinical outcomes in a subpopulation of adults with Morquio A syndrome: results from a long-term extension study of elosulfase alfa [J]. Orphanet Journal of Rare Diseases, 2017, 12(1).
[18] HENDRIKSZ C J, PARINI R, ALSAYED M D, et al. Long-term endurance and safety of elosulfase alfa enzyme replacement therapy in patients with Morquio A syndrome [J]. Molecular Genetics and Metabolism, 2016, 119(1-2): 131-43.
[19] BURTON B K, BERGER K I, LEWIS G D, et al. Safety and physiological effects of two different doses of elosulfase alfa in patients with morquio a syndrome: A randomized, double-blind, pilot study [J]. American Journal of Medical Genetics, Part A, 2015, 167(10): 2272-81.
[20] GUFFON N, CHOWDARY P, TELES E L, et al. Oral treatment for mucopolysaccharidosis VI: Outcomes of the first phase IIa study with odiparcil [J]. Journal of Inherited Metabolic Disease, 2022, 45(2): 340-52.
[21] HARMATZ P, GIUGLIANI R, SCHWARTZ I, et al. Enzyme replacement therapy for mucopolysaccharidosis VI: A phase 3, randomized, double-blind, placebo-controlled, multinational study of recombinant human N-acetylgalactosamine 4-sulfatase (recombinant human arylsulfatase B or rhASB) and follow-on, open-label extension study [J]. Journal of Pediatrics, 2006, 148(4): 533-.e.
[22] HARMATZ P, WHITLEY C B, WABER L, et al. Enzyme replacement therapy in mucopolysaccharidosis VI (Maroteaux-Lamy syndrome) [J]. Journal of Pediatrics, 2004, 144(5): 574-80.
[23] HARMATZ P R, GARCIA P, GUFFON N, et al. Galsulfase (Naglazyme®) therapy in infants with mucopolysaccharidosis VI [J]. Journal of Inherited

Metabolic Disease, 2014, 37(2): 277-87.
