# Supplementary material for: Efficacy of different treatment strategies in patients with mucopolysaccharidosis: a systematic review and network meta-analysis of randomized controlled trials
Source: Orphanet J Rare Dis. 2025 May 2;20:211. doi: 10.1186/s13023-025-03735-y (PMC12049060; doi:10.1186/s13023-025-03735-y)
Supplement: Supplementary file 4 — Supplementary Material 4: Appendix 4. Network plots for each outcome. [file 13023_2025_3735_MOESM4_ESM.pdf]

**Appendix 4 Network plots for each outcome**

The size of the circle in each network is proportional to the number of participants randomly assigned to the treatment comparison. The width of each line is proportional to the number of trials comparing the two connected treatments. When a line is absent, this indicates that there were no head-to-head trials of the corresponding treatments reporting the outcome of interest.

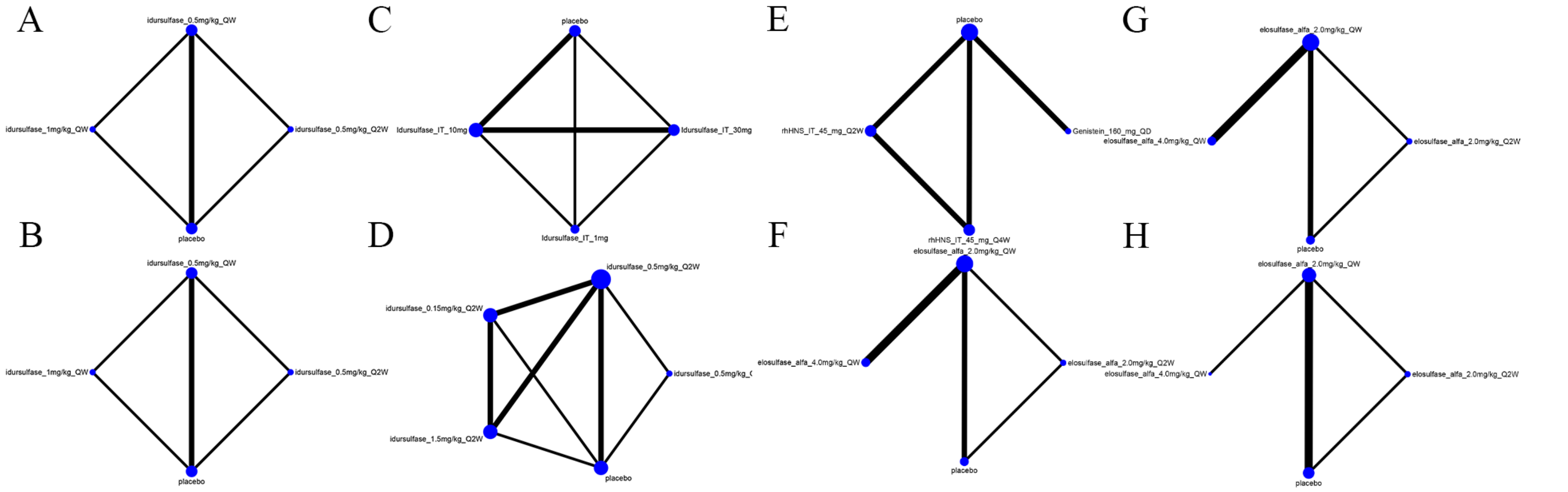


Figure Network plots for each outcome. (A) represents 6MWT in patients with MPS II; (B) represents FVC in patients with MPS II; (C) represents CSF GAG in patients with MPS II; (D)represents liver volumes in patients with MPS II; (E) represents cognitive DQ score in patients with MPS III; (F) represents 6MWT in patients with MPS IV; (G) represents 3MSCT in patients with MPS IV; (H) represents uKS in patients with MPS IV.
