# Supplementary figures and images for: Efficacy of different treatment strategies in patients with mucopolysaccharidosis: a systematic review and network meta-analysis of randomized controlled trials
Source: Orphanet J Rare Dis. 2025 May 2;20:211. doi: 10.1186/s13023-025-03735-y (PMC12049060; doi:10.1186/s13023-025-03735-y)

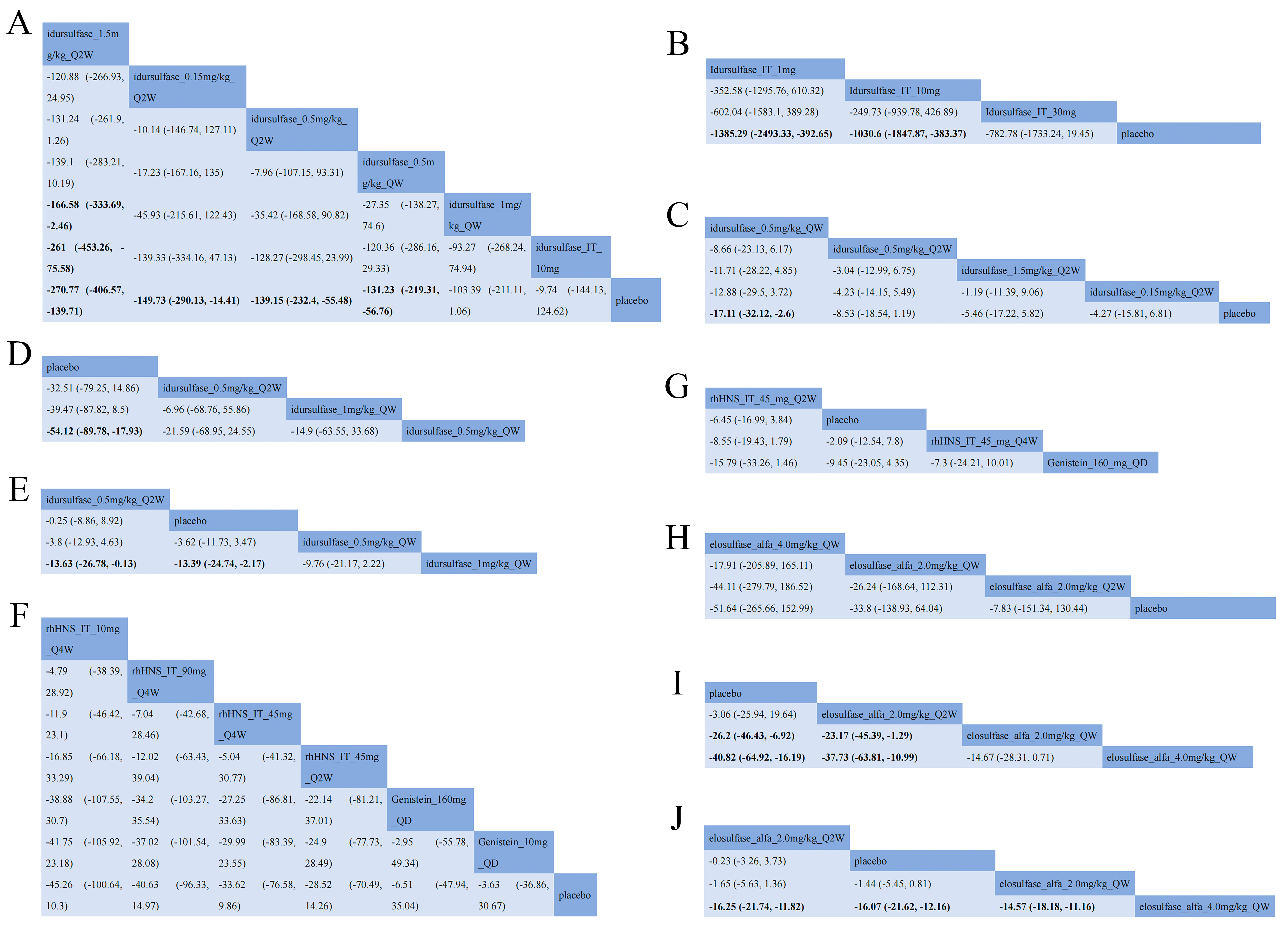

Supplement: Supplementary file 5 — Supplementary Material 5: Appendix 5. League tables. The columns present the row drug class compared to the column drug class. The rows present the row drug class compared to the column drug class. The effect estimates are expressed as mean ± standard deviation and 95% confidence intervals. Significant results are in bold. A. UGAG difference in patients with MPS II. B. CSF GAG difference in patients with MPS II. C. Liver volumes difference in patients with MPS II. D. 6MWT difference in patients with MPS II. E. FVC difference in patients with MPS II. F. UGAG difference in patients with MPS III. G. Cognitive DQ score difference in patients with MPS III. H. UKS difference in patients with MPS IV. I. 6MWT difference in patients with MPS IV. J. 3MSCT difference in patients with MPS IV. [file 13023_2025_3735_MOESM5_ESM.png]

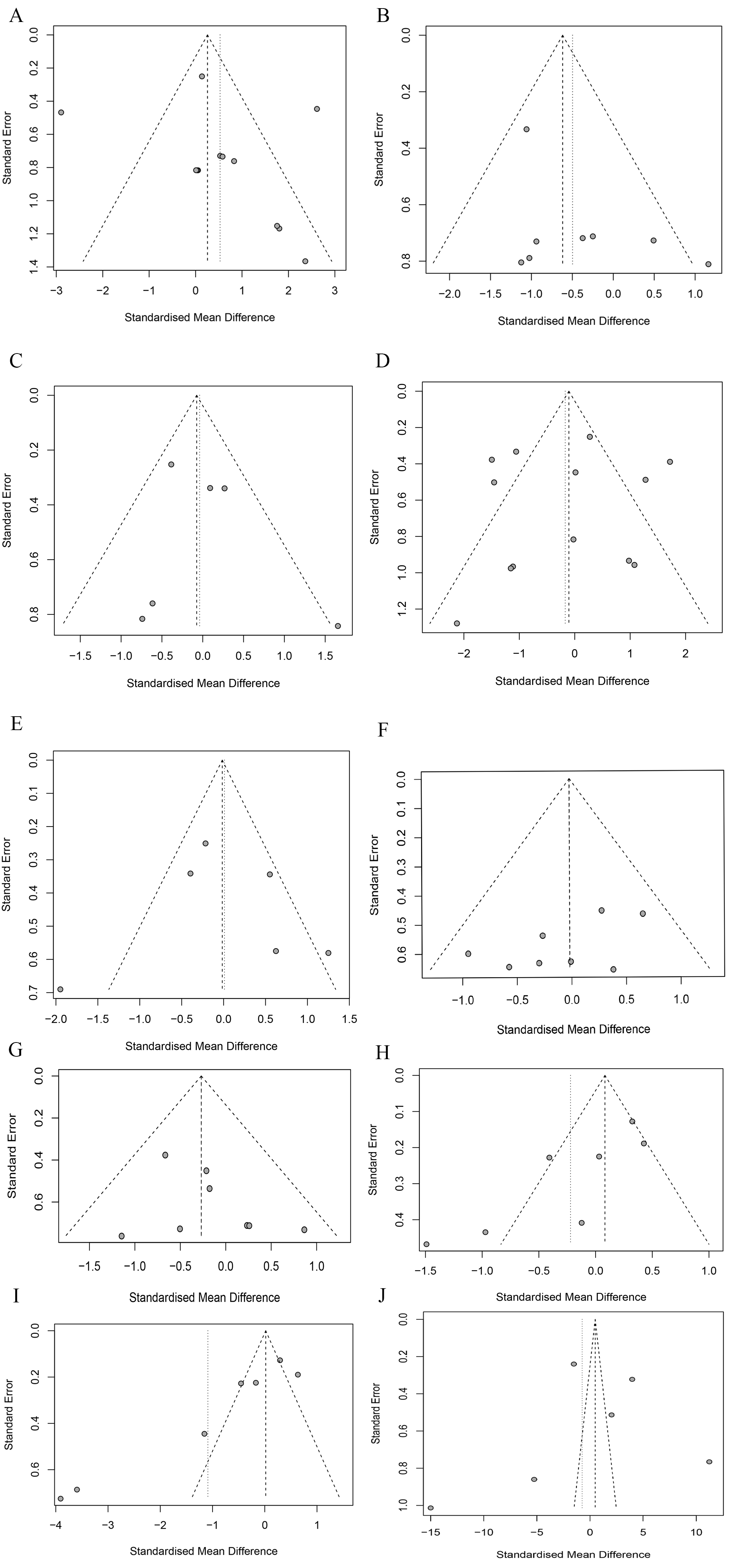

Supplement: Supplementary file 7 — Supplementary Material 7: Appendix 7. The funnel plot of enrolled trials. A, liver volumes reduction in patients with MPS II. B, CSF GAG in patients with MPS II. C, FVC in patients with MPS II. D, uGAG in patients with MPS II. E, 6MWT in patients with MPS II. F, cognitive DQ score in patients with MPS III. G, uGAG in patients with MPS III. H, 6MWT in patients with MPS IV. I, 3MSC in patients with MPS IV. J, uKS in patients with MPS IV. [file 13023_2025_3735_MOESM7_ESM.png]

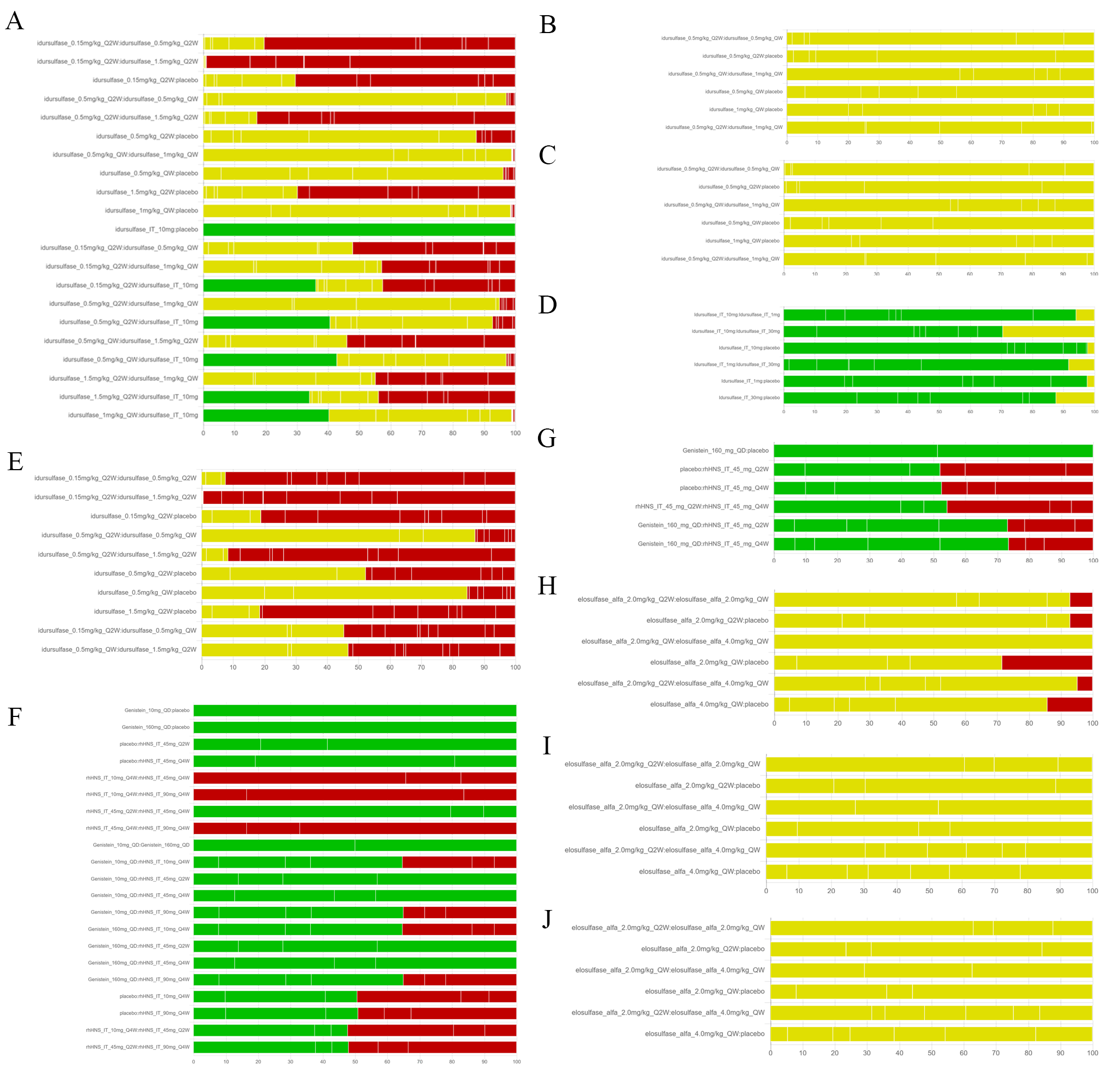

Supplement: Supplementary file 8 — Supplementary Material 8: Appendix 8. Evaluation of the Certainty of evidence Using CINEMA Framework, mixed evidence. A. UGAG quality in MPS II. B. 6MWT quality in MPS II. C. FVC quality in MPS II. D. CSF GAG quality in MPS II. E. Liver volumes quality in MPS II. F. UGAG quality in MPS III. G. Cognitive DQ score quality in MPS III. H. UKS quality in MPS IV. I. 3MSCT quality in MPS IV. J. 6MWT quality in MPS IV. [file 13023_2025_3735_MOESM8_ESM.png]
