# Supplementary material for: Efficacy of different treatment strategies in patients with mucopolysaccharidosis: a systematic review and network meta-analysis of randomized controlled trials
Source: Orphanet J Rare Dis. 2025 May 2;20:211. doi: 10.1186/s13023-025-03735-y (PMC12049060; doi:10.1186/s13023-025-03735-y)
Supplement: Supplementary file 6 — Supplementary Material 6: Appendix 6. Model fit. [file 13023_2025_3735_MOESM6_ESM.pdf]

Appendix 6 Model fit

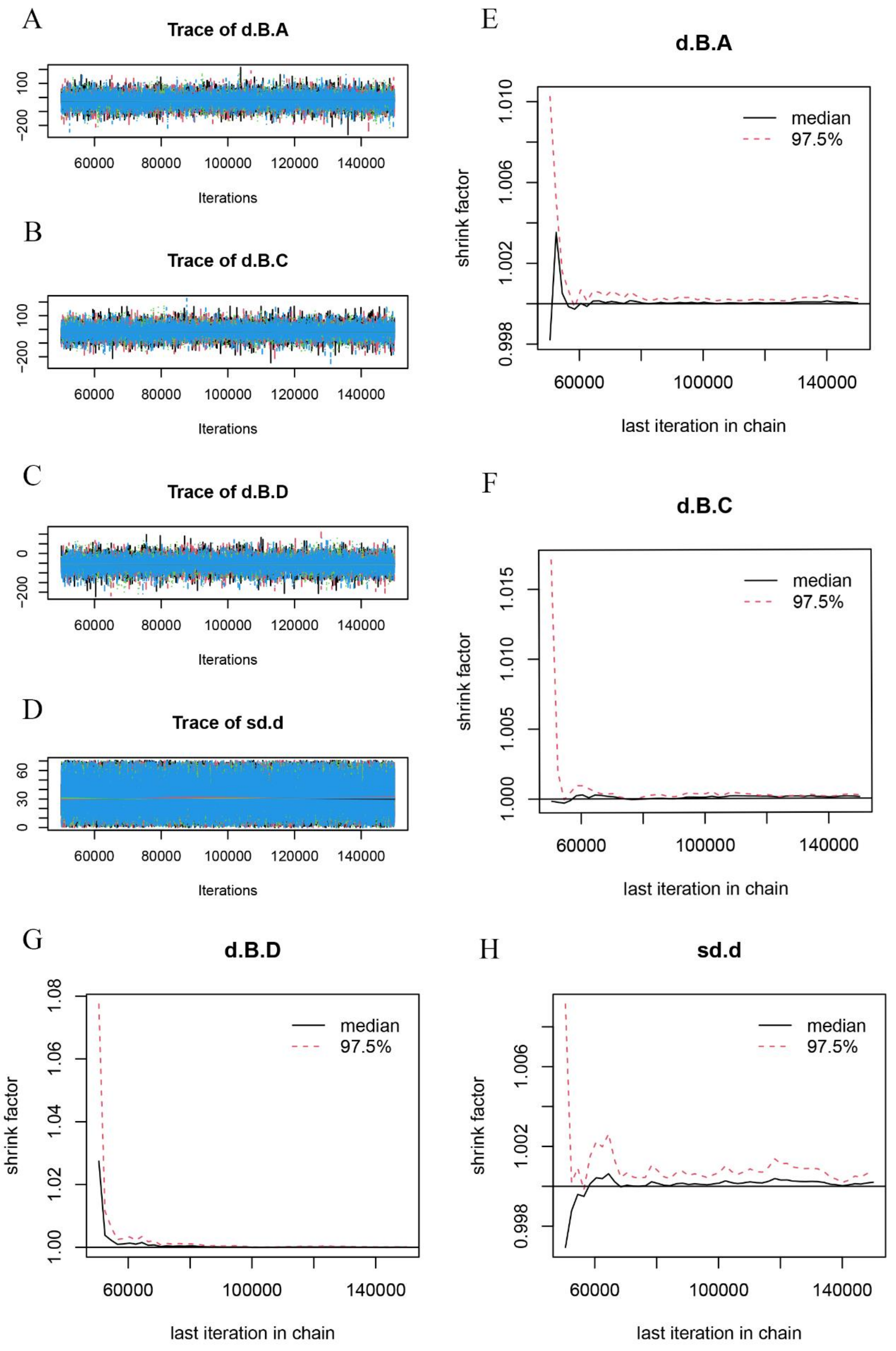

Figure S1 Results of convergence analysis for 6MWT in patients with MPS II. (A)-(D) are the trace plots and (E)-(H) are the Brooks-Gelman-Rubin diagnosis plots.

(A) and (E) represents idursulfase treatment with 0.5mg/kg every other week vs idursulfase treatment with 0.5mg/kg/week; (B) and (F) represents idursulfase treatment with 1mg/kg/week vs idursulfase treatment with 0.5mg/kg/week; (C) and (G) represents placebo vs idursulfase treatment with 0.5mg/kg/week; (D) and (H) represents overall comparison.

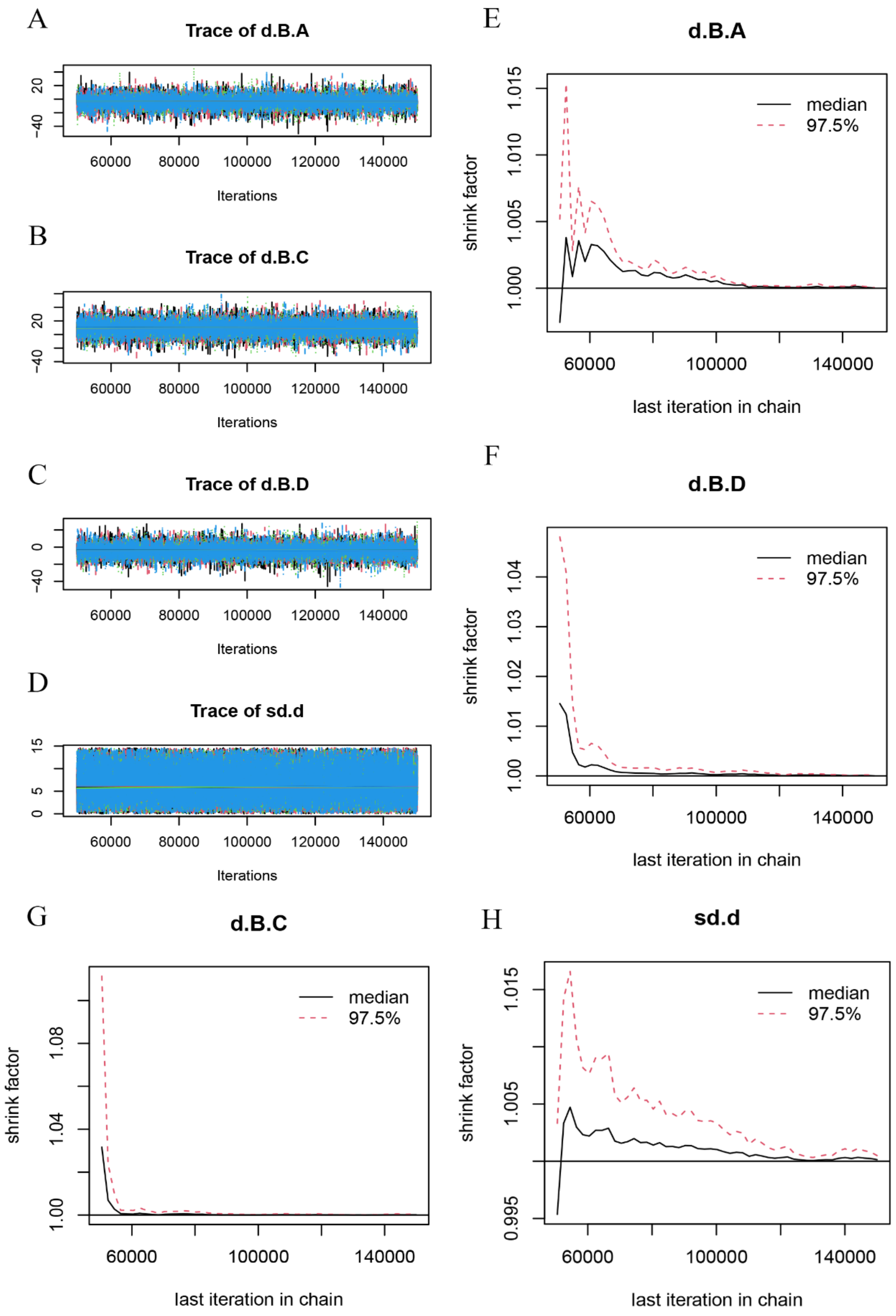

Figure S2 Results of convergence analysis for FVC in patients with MPS II. (A)-(D) are the trace plots and (E)-(H) are the Brooks-Gelman-Rubin diagnosis plots.(A) and (E) represents idursulfase treatment with 0.5mg/kg every other week vs idursulfase treatment with 0.5mg/kg/week; (B) and (G) represents idursulfase treatment with 1mg/kg/week vs idursulfase treatment with 0.5mg/kg/week; (C) and (F) represents placebo vs idursulfase treatment with 0.5mg/kg/week; (D) and (H) represents overall comparison.

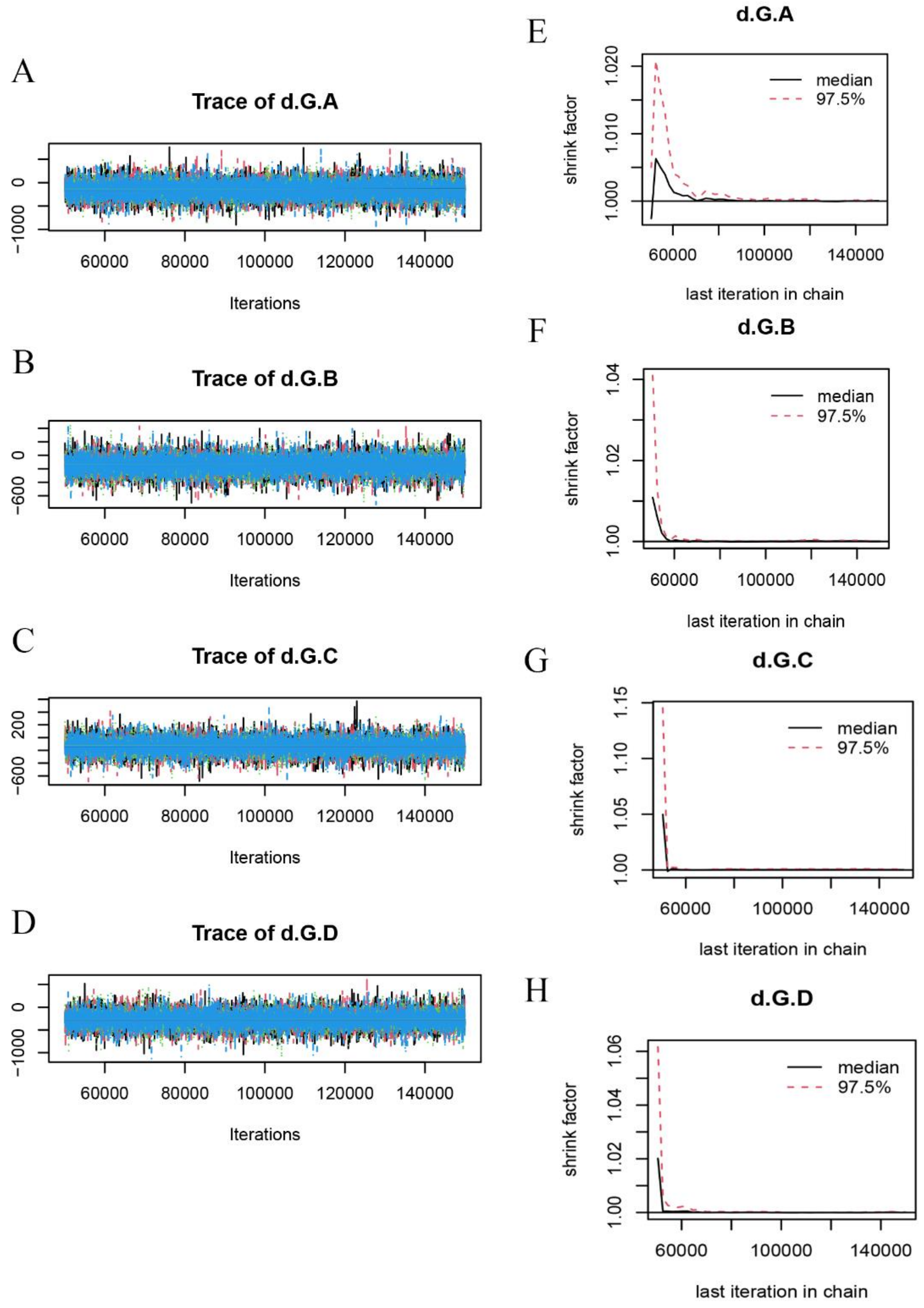

Figure S3a Results of convergence analysis for uGAG in patients with MPS II. (A)-(D) are the trace plots and (E)-(H) are the Brooks-Gelman-Rubin diagnosis plots. (A) and (E) represents idursulfase treatment with 0.15mg/kg every other week vs

placebo; (B) and (F) represents idursulfase treatment with 0.5mg/kg every other week vs placebo; (C) and (G) represents placebo vs idursulfase treatment with 0.5mg/kg/week; (D) and (H) represents idursulfase treatment with 1.5mg/kg every other week vs placebo.

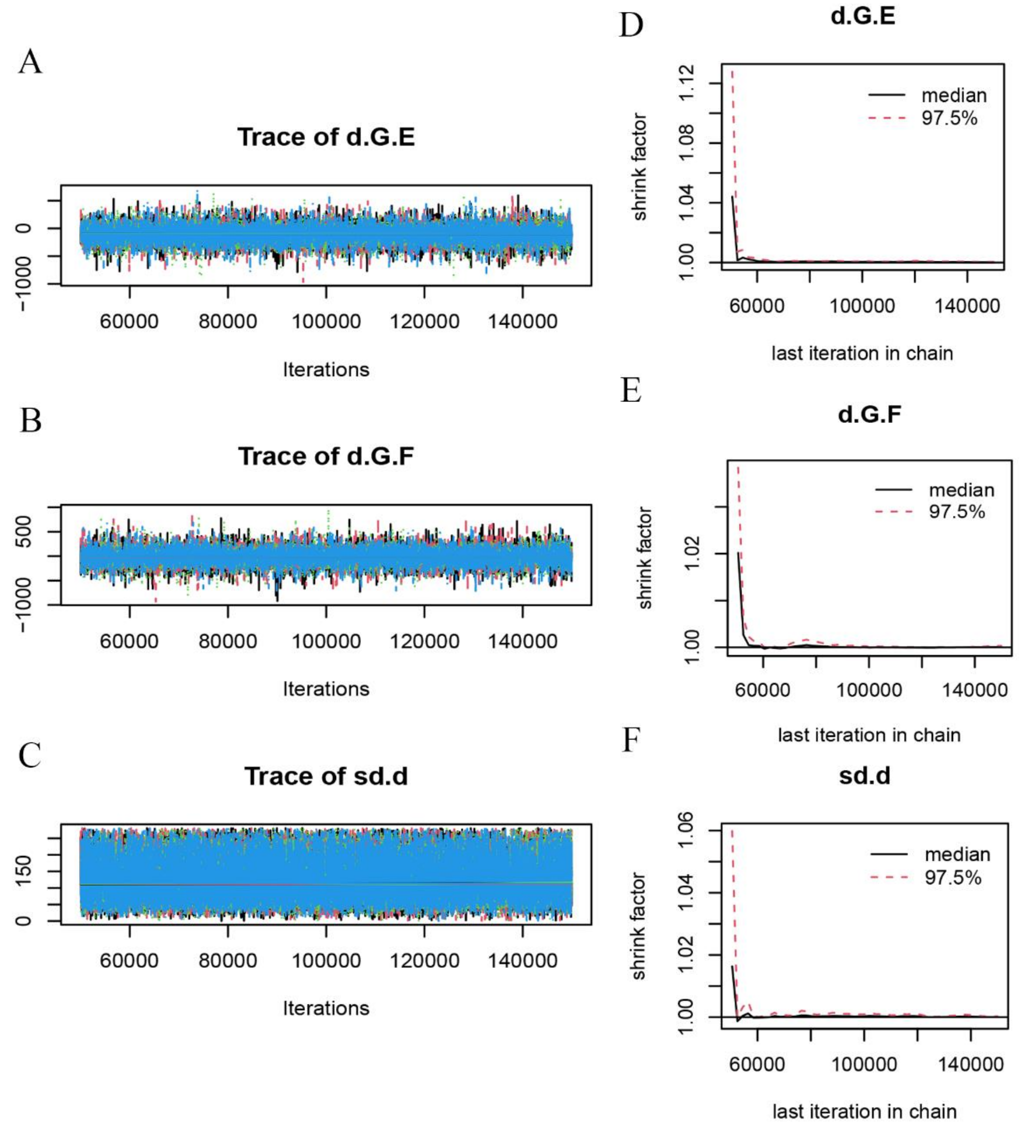

Figure S3b Results of convergence analysis for uGAG in patients with MPS II. (A)-(C) are the trace plots and (D)-(F) are the Brooks-Gelman-Rubin diagnosis plots. (A) and (D) represents idursulfase treatment with 1mg/kg/week vs placebo; (B) and (E) represents idursulfase IT treatment with 10mg vs placebo; (C) and (F) represents overall comparison.

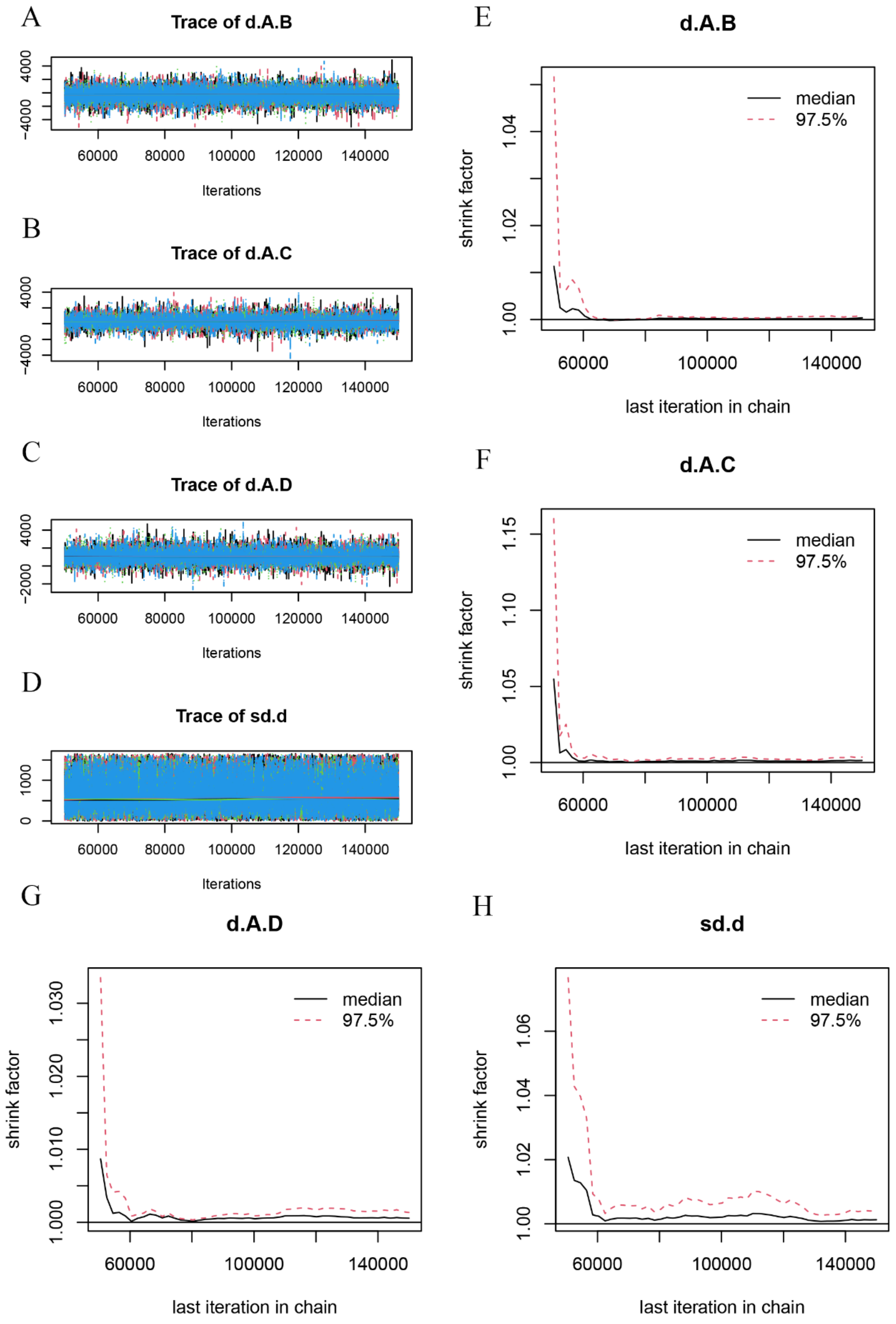

Figure S4 Results of convergence analysis for CSF GAG in patients with MPS II. (A)-(D) are the trace plots and (E)-(H) are the Brooks-Gelman-Rubin diagnosis plots. (A) and (E) represents idursulfase IT treatment with 10mg vs idursulfase IT treatment

with 1mg; (B) and (F) represents idursulfase IT treatment with 10mg vs idursulfase IT treatment with 30mg; (C) and (G) represents placebo vs idursulfase IT treatment with 10mg; (D) and (H) represents overall comparison.

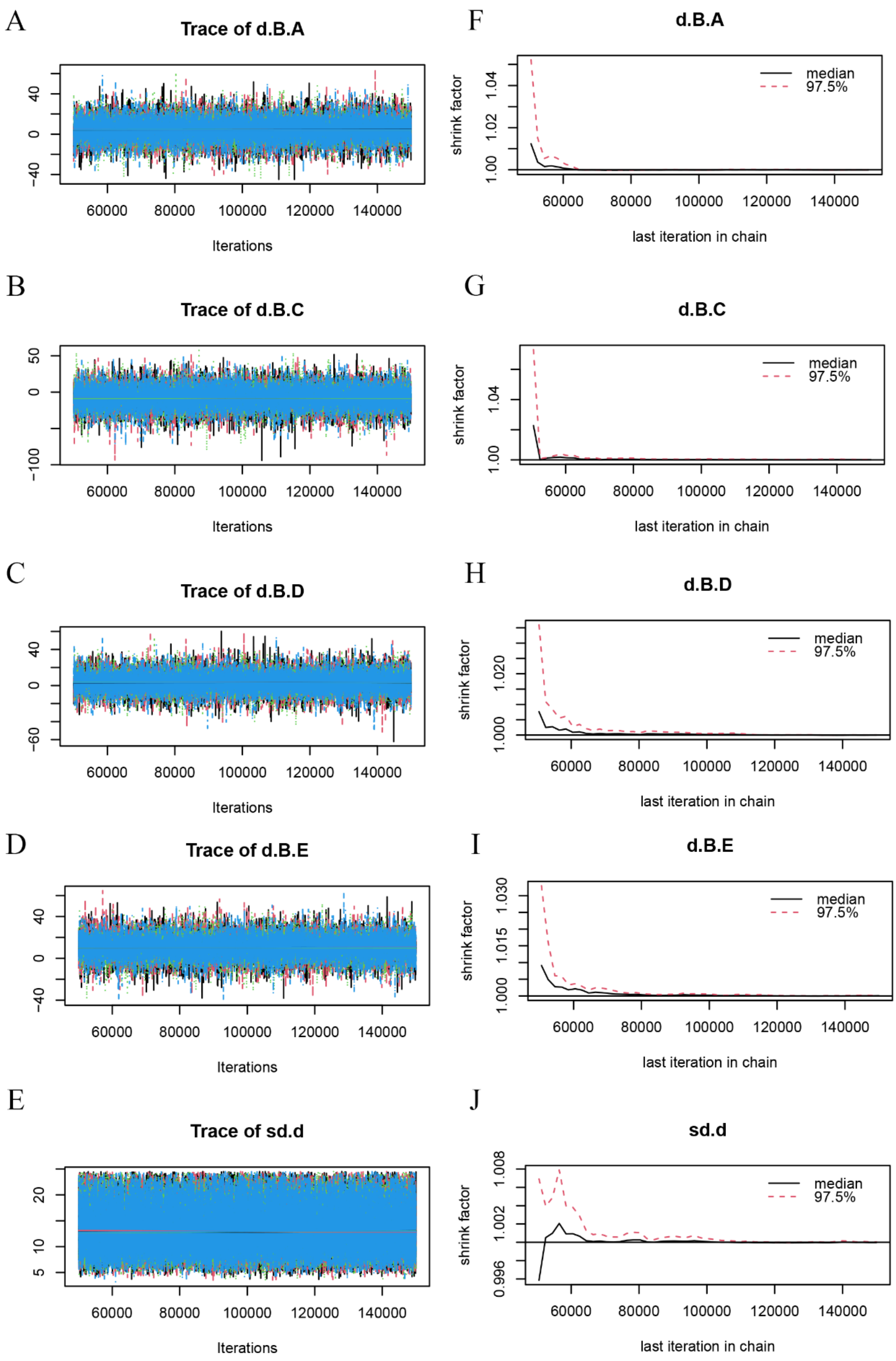

Figure S5 Results of convergence analysis for liver volume in patients with MPS II. (A)-(E) are the trace plots and (F)-(J) are the Brooks-Gelman-Rubin diagnosis plots. (A) and (F) represents idursulfase treatment with 0.15mg/kg every other week vs

idursulfase treatment with 0.5mg/kg every other week; (B) and (G) represents idursulfase treatment with 0.5mg/kg every other week vs idursulfase treatment with 0.5mg/kg/week; (C) and (H) represents idursulfase treatment with 0.5mg/kg every other week vs idursulfase treatment with 1.5mg/kg every other week; (D) and (I) represents idursulfase treatment with 0.5mg/kg every other week vs placebo;(E) and (J) represents overall comparison.

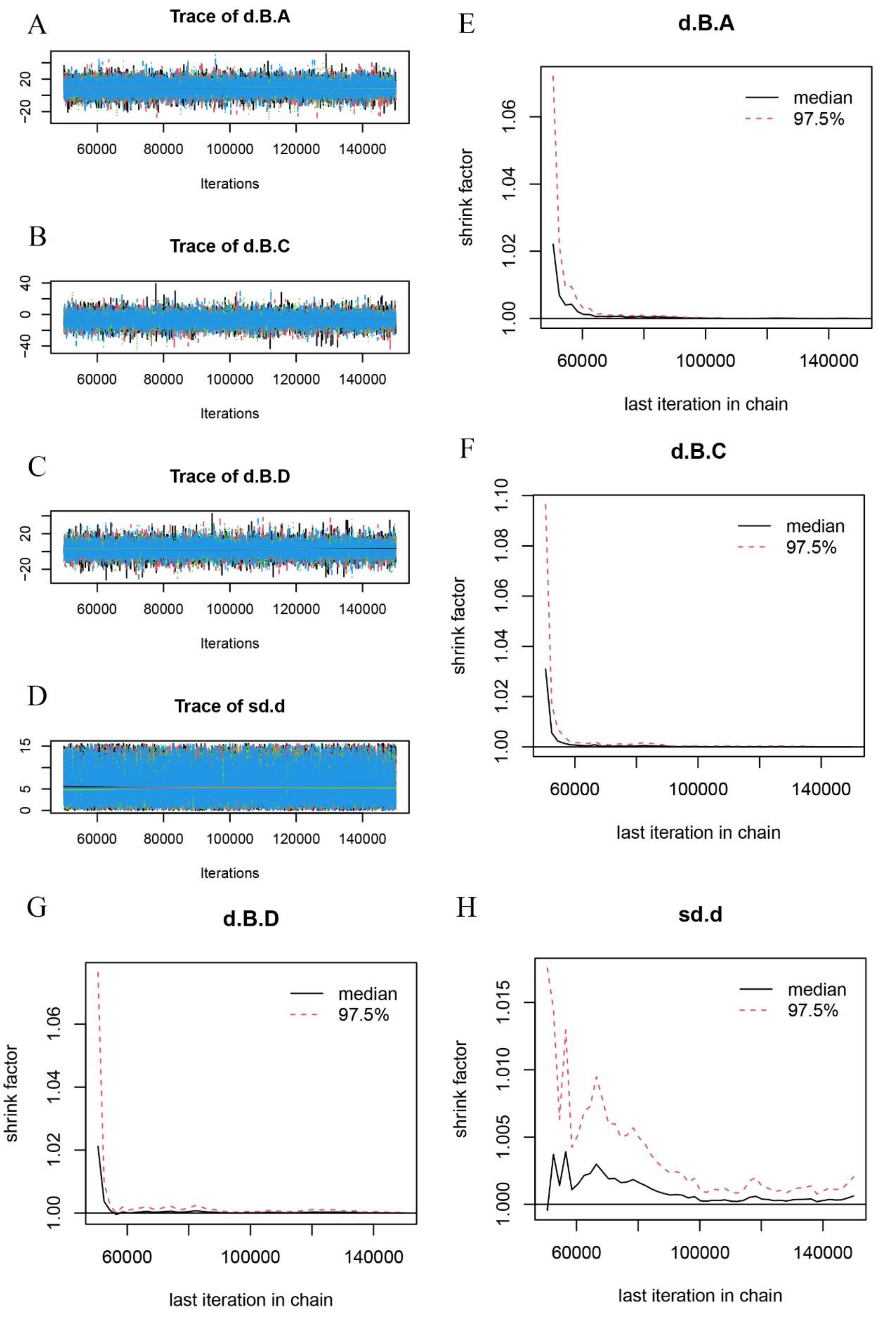

Figure S6 Results of convergence analysis for cognitive DQ score in patients with MPS III. (A)-(D) are the trace plots and (E)-(H) are the Brooks-Gelman-Rubin diagnosis plots. (A) and (E) represents Genistein 160mg vs placebo; (B) and (F) represents rhHNS IT treatment with 45mg every other week vs placebo; (C) and (G)

represents rhHNS IT treatment with 45mg every four weeks vs placebo; (D) and (H) represents overall comparison.

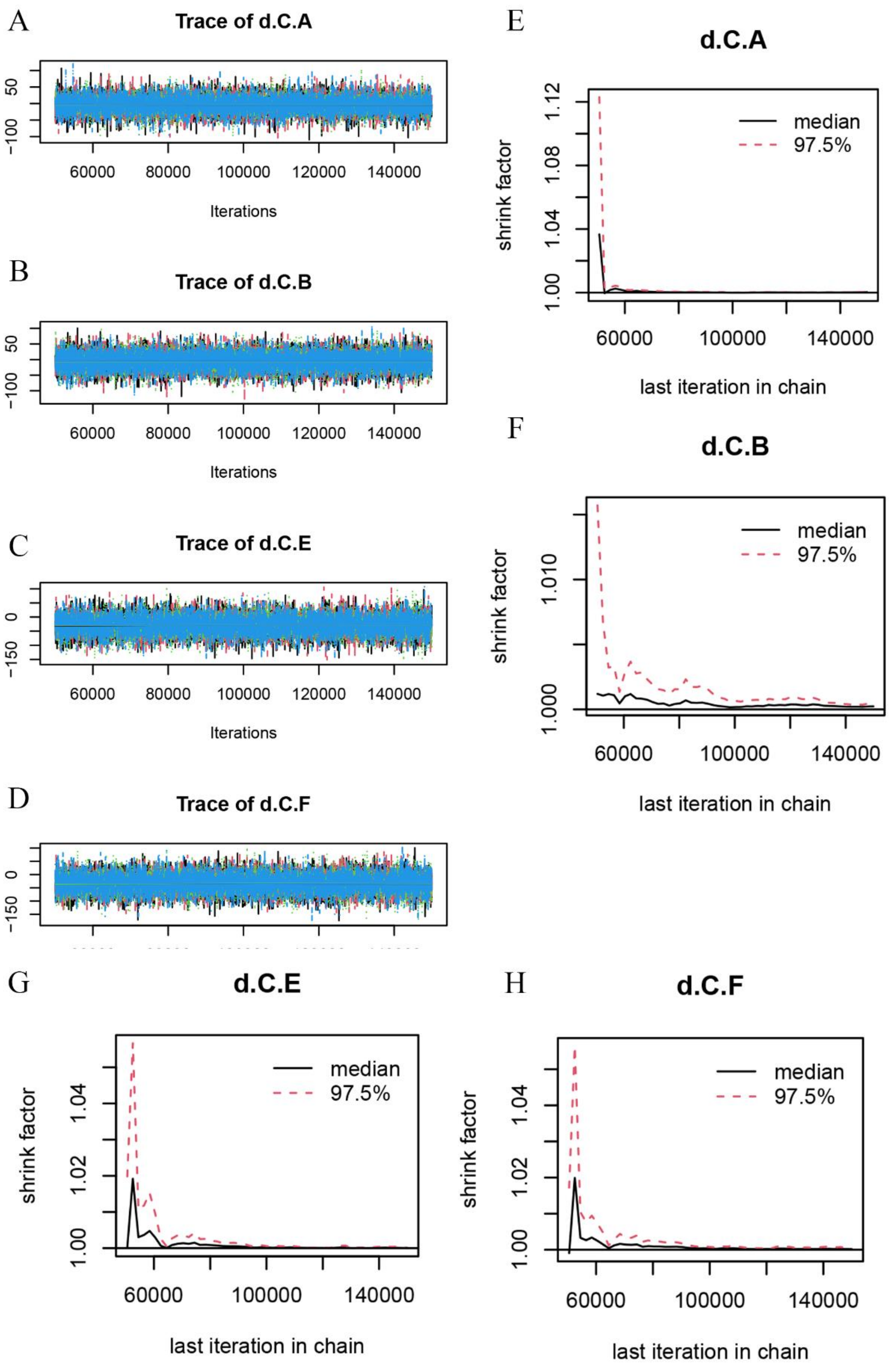

Figure S7a Results of convergence analysis for uGAG in patients with MPS III.

(A)-(D) are the trace plots and (E)-(H) are the Brooks-Gelman-Rubin diagnosis plots. (A) and (E) represents Genistein 10mg vs placebo; (B) and (F) represents Genistein 160mg vs placebo; (C) and (G) represents rhHNS IT treatment with 45mg every other week vs placebo; (D) and (H) represents rhHNS IT treatment with 45mg every four weeks vs placebo.

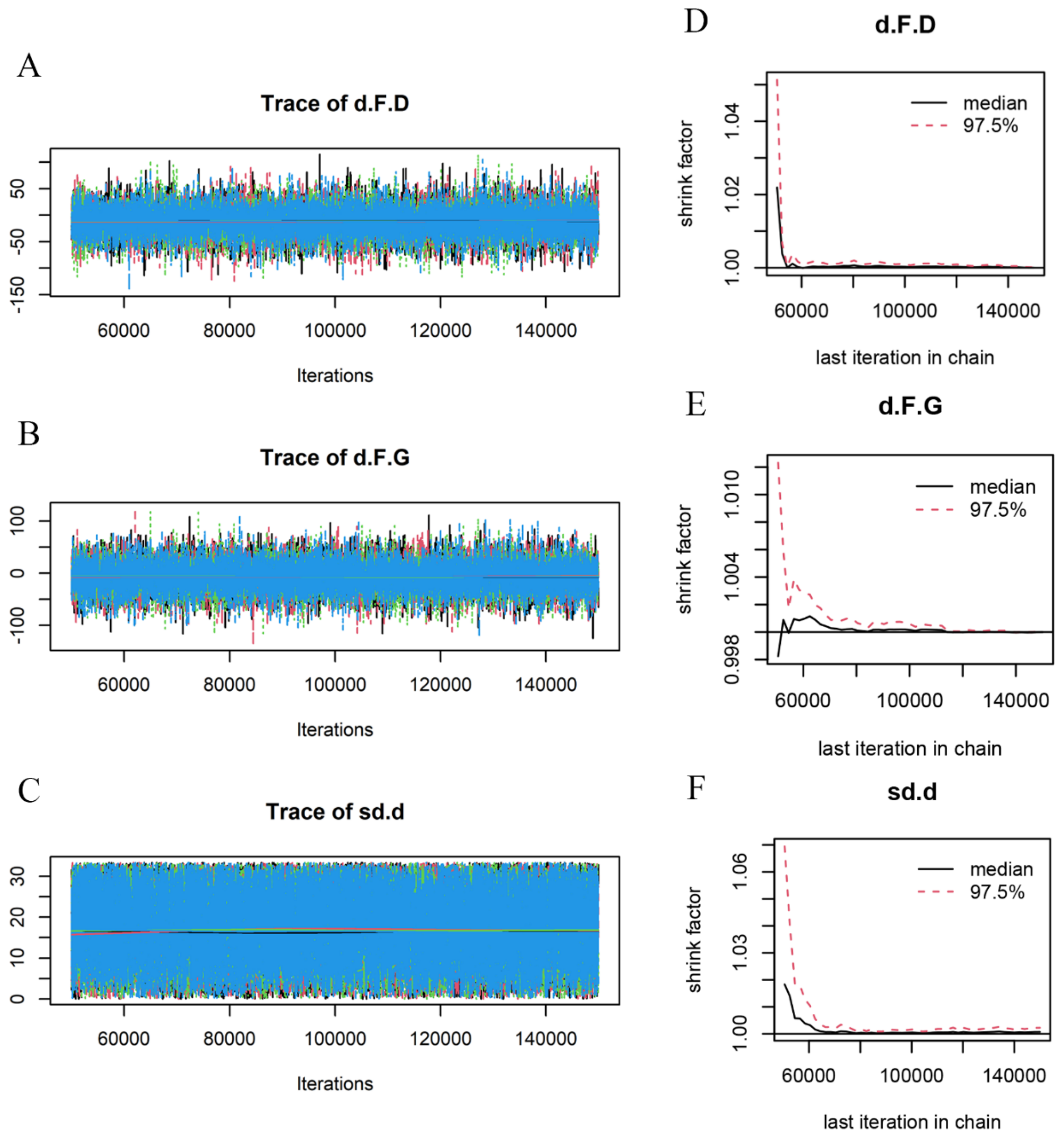

Figure S7b Results of convergence analysis for uGAG in patients with MPS III. (A)-(C) are the trace plots and (D)-(F) are the Brooks-Gelman-Rubin diagnosis plots. (A) and (D) represents rhHNS IT treatment with 45mg every four weeks vs rhHNS IT treatment with 10mg every four weeks; (B) and (E) represents rhHNS IT treatment with 45mg every four weeks vs rhHNS IT treatment with 90mg every four weeks; (C) and (F) represents overall comparison.

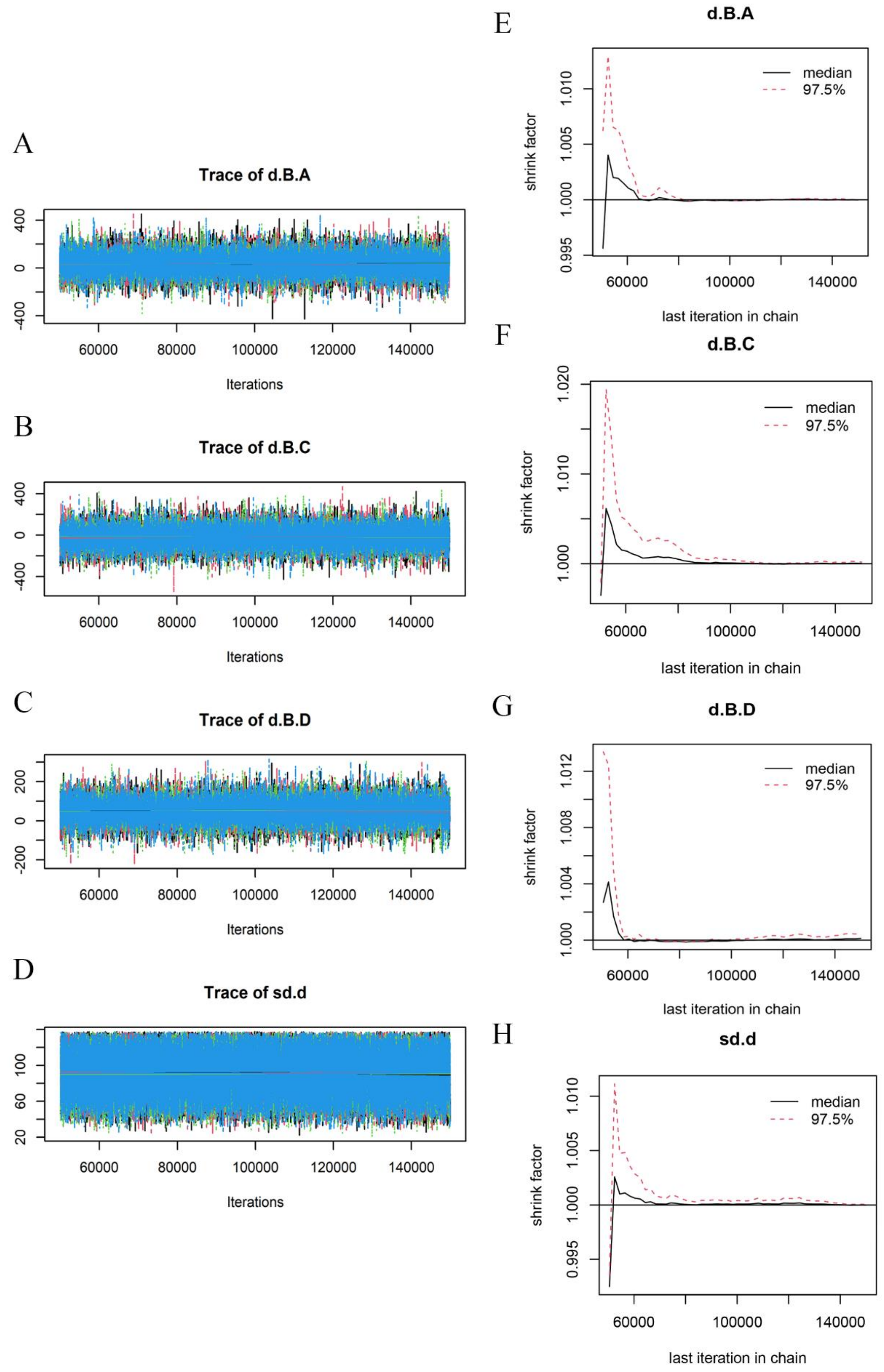

Figure S8 Results of convergence analysis for uKS in patients with MPS IV. (A)-(D) are the trace plots and (E)-(H) are the Brooks-Gelman-Rubin diagnosis plots. (A) and (E) represents elosulfase alfa treatment with 2.0mg/kg every other week vs

elosulfase alfa treatment with 2.0mg/kg/week; (B) and (F) represents elosulfase alfa treatment with 2.0mg/kg/week vs elosulfase alfa treatment with 4.0mg/kg/week; (C) and (G) represents elosulfase alfa treatment with 2.0mg/kg/week vs placebo; (D) and (H) represents overall comparison.

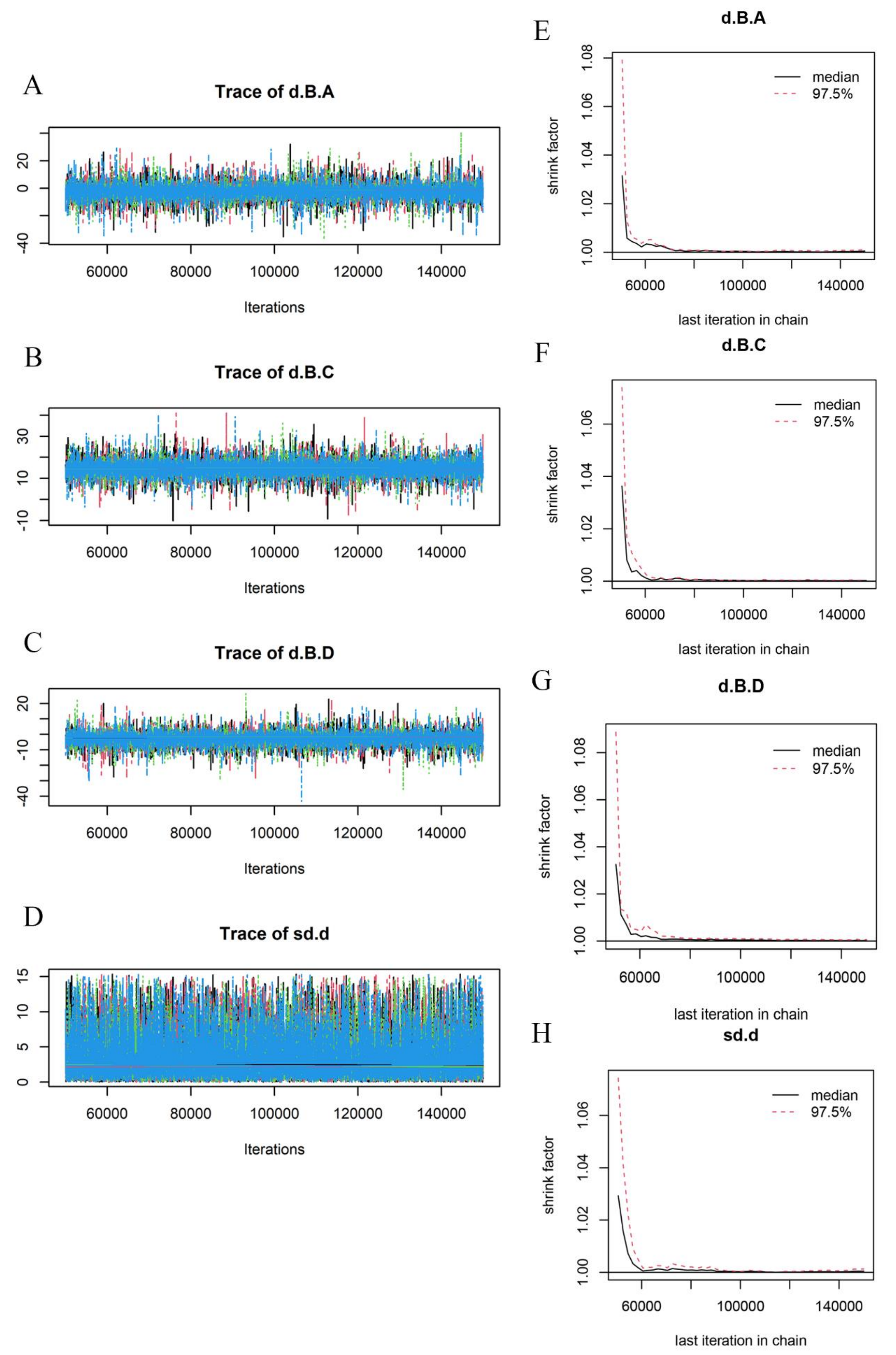

Figure S9 Results of convergence analysis for 3MSCT in patients with MPS IV. (A)-(D) are the trace plots and (E)-(H) are the Brooks-Gelman-Rubin diagnosis plots. (A) and (E) represents elosulfase alfa treatment with 2.0mg/kg every other week vs

elosulfase alfa treatment with 2.0mg/kg/week; (B) and (F) represents elosulfase alfa treatment with 2.0mg/kg/week vs elosulfase alfa treatment with 4.0mg/kg/week; (C) and (G) represents elosulfase alfa treatment with 2.0mg/kg/week vs placebo; (D) and (H) represents overall comparison.

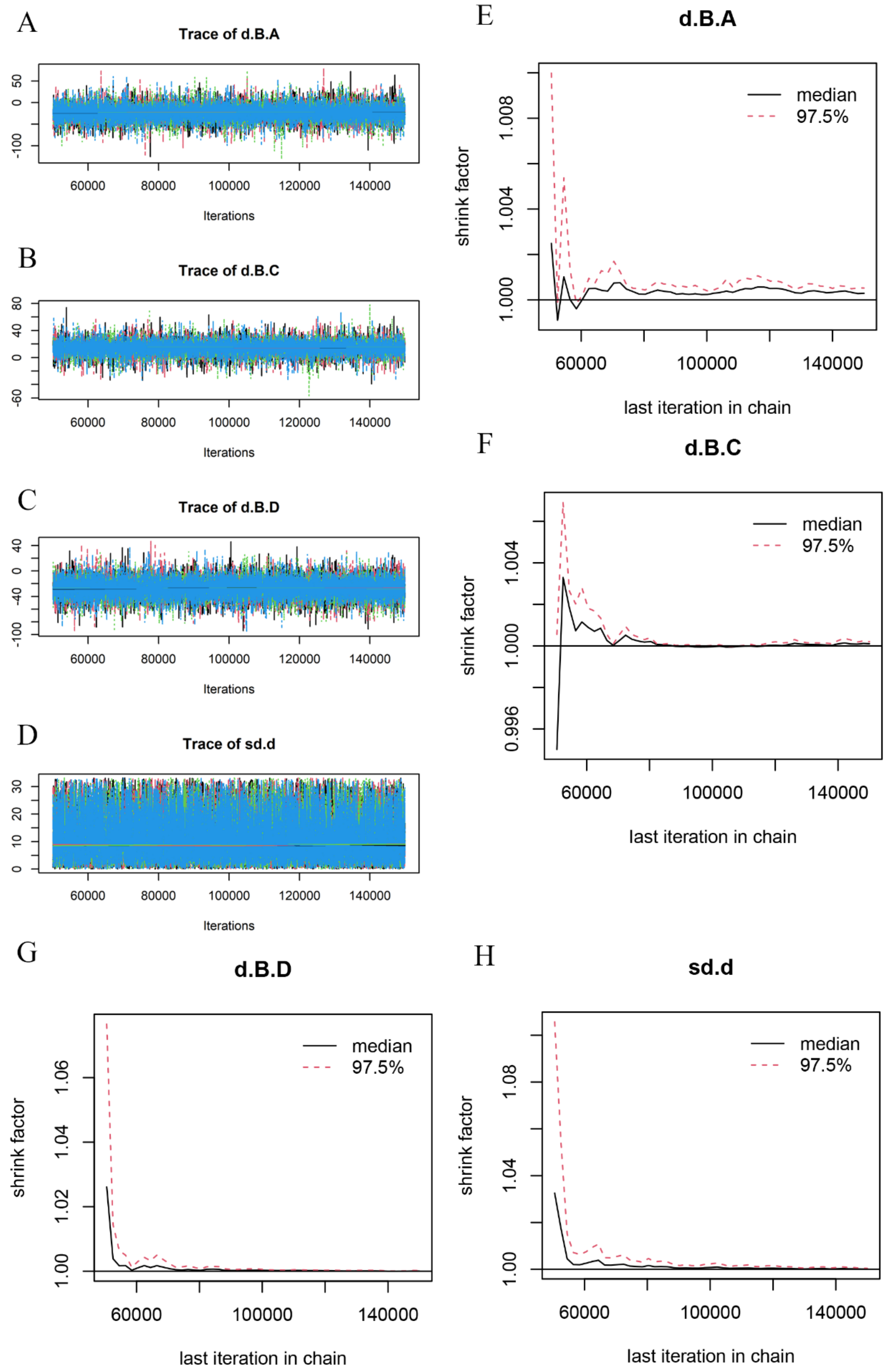

Figure S10 Results of convergence analysis for 6MWT in patients with MPS IV. (A)-(D) are the trace plots and (E)-(H) are the Brooks-Gelman-Rubin diagnosis plots. (A) and (E) represents elosulfase alfa treatment with 2.0mg/kg every other week vs

elosulfase alfa treatment with 2.0mg/kg/week; (B) and (F) represents elosulfase alfa treatment with 2.0mg/kg/week vs elosulfase alfa treatment with 4.0mg/kg/week; (C) and (G) represents elosulfase alfa treatment with 2.0mg/kg/week vs placebo; (D) and (H) represents overall comparison.
